# Similar Arbuscular Mycorrhizal Fungal Communities in 31 Durum Wheat Cultivars (*Triticum turgidum* L. var. durum) Under Field Conditions in Eastern Canada

**DOI:** 10.3389/fpls.2020.01206

**Published:** 2020-08-11

**Authors:** Franck Stefani, Sarah Dupont, Mario Laterrière, Ron Knox, Yuefeng Ruan, Chantal Hamel, Mohamed Hijri

**Affiliations:** ^1^ Ottawa Research and Development Centre of Agriculture and Agri-Food Canada, Ottawa, ON, Canada; ^2^ Département de Sciences Biologiques, Institut de Recherche en Biologie Végétale, Université de Montréal, Montréal, QC, Canada; ^3^ Quebec Research and Development Centre of Agriculture and Agri-Food Canada, Quebec, QC, Canada; ^4^ Swift Current Research and Development Centre of Agriculture and Agri-Food Canada, Swift Current, SK, Canada; ^5^ AgroBioSciences, Mohammed VI Polytechnic University, Ben Guerir, Morocco

**Keywords:** arbuscular mycorrhizal fungal communities, durum wheat cultivars, plant breeding, symbiosis, *Triticum turgidum* var. *durum*

## Abstract

Wheat is among the important crops harnessed by humans whose breeding efforts resulted in a diversity of genotypes with contrasting traits. The goal of this study was to determine whether different old and new cultivars of durum wheat (*Triticum turgidum* L. var. *durum*) recruit specific arbuscular mycorrhizal (AM) fungal communities from indigenous AM fungal populations of soil under field conditions. A historical set of five landraces and 26 durum wheat cultivars were field cultivated in a humid climate in Eastern Canada, under phosphorus-limiting conditions. To characterize the community of AMF inhabiting bulk soil, rhizosphere, and roots, MiSeq amplicon sequencing targeting the 18S rRNA gene (SSU) was performed on total DNAs using a nested PCR approach. Mycorrhizal colonization was estimated using root staining and microscope observations. A total of 317 amplicon sequence variants (ASVs) were identified as belonging to Glomeromycota. The core AM fungal community (i.e., ASVs present in > 50% of the samples) in the soil, rhizosphere, and root included 29, 30, and 29 ASVs, respectively. ASVs from the genera *Funneliformis*, *Claroideoglomus*, and *Rhizophagus* represented 37%, 18.6%, and 14.7% of the sequences recovered in the rarefied dataset, respectively. The two most abundant ASVs had sequence homology with the 18S sequences from well-identified herbarium cultures of *Funneliformis mosseae* BEG12 and *Rhizophagus irregularis* DAOM 197198, while the third most abundant ASV was assigned to the genus *Paraglomus*. Cultivars showed no significant difference of the percentage of root colonization ranging from 57.8% in Arnautka to 84.0% in AC Navigator. Cultivars were generally associated with similar soil, rhizosphere, and root communities, but the abundance of *F. mosseae*, *R. irregularis*, and *Claroideoglomus* sp. sequences varied in Eurostar, Golden Ball, and Wakooma. Although these results were obtained in one field trial using a non-restricted pool of durum wheat and at the time of sampling, that may have filtered the community in biotopes. The low genetic variation between durum wheat cultivars for the diversity of AM symbiosis at the species level suggests breeding resources need not be committed to leveraging plant selective influence through the use of traditional methods for genotype development.

## Introduction

Durum wheat (*Triticum turgidum* L. var. *durum* Desf.) is a major crop in Canada with an average annual production of 5.96 million tonnes from 2015 to 2019 (Statistics Canada, 2020), establishing Canada as the largest exporter of durum wheat in the world. The flour derived from durum grains is mostly used for the production of pasta, semolina bourghul, and breads. Durum wheat originates from the Fertile Crescent and was the major cultivated form of tetraploid wheat during the Hellenistic period ca. 2300 BP (Feldman and Kislev, 2008). It was introduced into Western Canada in the late 19^th^ century where 80% of the production occurred in the Brown and Dark Brown soil zones (McCaig and Clarke, 1995). Farmers increased cultivation of durum wheat in Western Canada in the 1960s because it was less susceptible to stem rust compared to bread wheat varieties cultivated at that time. The first developed cultivar in Canada was Stewart 63 released in 1963. McCaig and Clarke (1995) estimated that the development of new cultivars through the Canadian durum breeding programs for the period 1960 to 1990 increased yields by about 25% compared to foreign cultivars available prior to Stewart 63. Gluten content, cadmium concentration, resistance to fungal pathogens (*Fusarium* head blight, leaf, and stem rust) and insect pests (wheat stem sawfly, wheat midge) were the main traits considered for developing new varieties (Dexter, 2008; Clarke et al., 2010).

The selection pressure applied in the 20^th^ century on the new varieties of durum wheat was therefore driven by commercial purposes and high performance in high input systems (fertilizers, pesticides). For a long time, plants were considered as autonomous individuals and, as a consequence, breeding approaches completely overlook the complex microbial context of the soil environment in which crops grow. More specifically, breeding approaches do not take into account the performance of the newly developed varieties to recruit root mutualists.

The association of roots with microorganisms relies on intricate molecular crosstalk which results from long-term co-evolution between plant hosts and microbial partners (Lambers et al., 2009). Modification of the root exudates following domestication and breeding can shape different microbial communities. Selective breeding in wild emmer, domesticated emmer, and modern durum wheat has triggered changes in root exudate composition (Iannucci et al., 2017). Less is known on the effect of selective breeding on the microbial associations of durum than on common wheat (*Triticum aestivum* L.), its hexaploid relative. High-throughput sequencing of rhizospheric bacterial communities associated with different winter wheat cultivars showed a line effect on the structure of the bacterial communities, suggesting that these communities could be manipulated by wheat breeding (Donn et al., 2015; Mahoney et al., 2017).

Among beneficial root associates, arbuscular mycorrhizal fungi (phylum Glomeromycota) coevolve with plants since ~980 Ma – 600 Ma. Arbuscular mycorrhizal (AM) fungi colonize the root system of 72% of vascular plants (Brundrett and Tedersoo, 2018) where they form highly branched fungal structures called arbuscules (Smith and Read, 2010). Arbuscules are the sites for nutrient exchanges between both partners. AM symbionts obtain plant carbon and, in exchange, release mineral nutrients absorbed from the soil. Despite their limited species richness (334 species described so far, www.amf-phylogeny.com), AM fungi, together with their associated microbiota, provide a range of essential services, from drought stress mitigation and disease prevention, to plant nutrient and water uptake, and the maintenance of biological soil fertility (Gianinazzi et al., 2010; Turrini et al., 2018). Fine tuning the interaction between the naturally occurring AM fungal communities and crop plants through plant breeding and appropriate agronomy, could improve the sustainability of agroecosystems (Bakker et al., 2012; Gan et al., 2015; Hijri, 2016).

Wheat has long been recognized as a crop with mixed responses to AM fungi, from negative, neutral, to positive effects. Hetrick et al. (1993) found strong dependence of winter wheat on mycorrhiza in cultivars released prior to 1950, but more variable responses in recently released cultivars. The authors suggested that cultivars released after 1950 had reduced dependence on mycorrhizae due to breeding performed under high fertility conditions. The impact of breeding on the mycorrhiza of durum wheat is less clear. In a ‘proof of concept’ experiment conducted under greenhouse conditions by the Canadian Government durum wheat breeding program (Singh et al., 2012), plant growth response to the model AM fungus *R. irregularis* DAOM 197198 varied among five cultivars (AC Morse, Commander, DT 710, Strongfield, Mongibelllo). Then, a thorough examination of the AM symbiosis formed between *R. irregularis* DAOM 197198 and five landraces and 27 modern cultivars (Canadian historical set) revealed that breeding had inconsistent effects on mycorrhiza development in durum wheat under greenhouse conditions. It led to the identification of cultivars with unimproved patterns of regulation of symbiotic development (e.g.: Commander, AC Pathfinder), and in a few cases, to cultivars (e.g.: Hercules, Wascana, Eurostar) with crippled regulation and poor plant performance in soil with high fertility (Ellouze et al., 2016). In a study that investigated the impact of these 32 cultivars on the structure of the AM fungal community in two field trials in the Canadian Prairies (Swift Current and Regina), Ellouze et al. (2018) reported significantly different relative abundance of the genus *Paraglomus* in the cultivars Ramsey (11%) and Strongfield (93.7%) and a significant effect of the cultivars on the structure of the AM fungal community in the rhizosphere, but not in the roots. However, this study was performed in the semiarid zone of Canadian prairie where moisture shortage could mask possible selective effects of cultivars on the AM fungal communities colonizing plant roots. Variation in soil moisture, the factor shaping the prairie ecosystems and more dramatically so the semiarid prairie (Hamel et al., 2006), was a confounding influence in this study. Moreover, the sequencing depth per sample was low with an average of 168 AM fungal pyrosequences per sample.

In order to overcome the abovementioned pitfalls and to discern possible differences in AM fungal community composition and root colonization percentages between genetically diverse durum wheat cultivars, a field trial seeded with five landraces and 26 cultivars released at different times in the history of durum wheat breeding was set up under a humid climate in Eastern Canada and the AM fungal community was thoroughly characterized by high-throughput sequencing. Based on the results from previous studies, we hypothesised that different field grown durum wheat cultivars associate with distinct AM fungal communities. The V3-V4 region of the nuclear 18S rRNA gene of AM fungi was sequenced to describe AM fungal communities located in bulk soil, rhizosphere soil, and roots, at anthesis.

## Materials and Methods

### Experimental Field-Trial

The experimental field was set up in 2016 nearby the city of Lévis (Québec, Canada, GPS coordinates: 46°47′40′′N; 71°08′05′′W). The region is featured by a growing season of 140 to 150 days, a cool and humid climate with average temperatures of 12.5°C, 16.9°C, and 19.1°C in May, June, and July, respectively, according to the Environment Canada weather station (https://climate.weather.gc.ca) located at 3.5 km from the experimental field. These temperatures are similar to those recorded for the same months during the period 1981 to 2010 (11.0°C, 16.5°C, and 19.3°C).

The soil was a well-drained Saint-André gravelly loam (fragic, humo-ferric podzol or mixed, frigid typic dystrochrept, Soil Classification Working Group, 1998). Physical and chemical properties of soil are provided in **Table S1** as supplementary material. The field was previously used to grow switchgrass (*Panicum virgatum* L.) in 2014 to 2015. Glyphosate-Roundup® was applied, and the field was tilled in fall 2015. Harrowing and fertilization were carried out on May 11, 2016. The plots at time of sowing received 90 kg/ha of nitrogen (N) as calcium ammonium nitrate (27-0-0) and 37 kg/ha potassium (K) as potassium chloride (0-0-60). Phosphorous (P) fertilization was not applied in order to make P resource a limiting factor to favour the mycorrhizal association. Five landraces and 26 durum wheat cultivars (details about each cultivar is provided in **Table S2**) were seeded at a density of 118 seeds/m^2^ using a 4-row cereal plot seeder on 12 May 2016. Seeds were obtained from the collection at Agriculture and Agri-Food Canada, Swift Current, SK. Each plot was 1 m × 1.7 m and four rows per plot were seeded with one out of the 31 cultivars. DyVel® herbicide (Dicamba) was applied at 1.25 L/ha on 7 June 2016 for weed control.

The field trial was arranged in a randomized complete block design, with four blocks, 31 cultivars per block, representing a total of 124 plots (**Figure S1**). Each block was layered in two rows of plots and a guard plot was seeded with cultivar AAC Cabri at both ends of each row.

### Field Sampling

To characterize the AM fungal community associated with durum wheat, three biologically relevant compartments were sampled: bulk soil, rhizosphere, and roots. Sampling was performed at anthesis, on July 8 and 12, 2016, as follows: six plants were randomly selected from within rows 2 and 3 of each plot and dug out with a spade. The aboveground portion of each plant was cut off and discarded, and the root system was stored at 4°C in a cooler in the field and then at 4°C in a laboratory fridge until processing. Six soil cores were collected between rows 3 and 4 using a soil probe (2.5 cm in diameter, 15 cm long). For each plot, the six soil cores were sieved (mesh size 2 mm) and combined into a single composite sample. The root system of each of the six plants per plot was gently shaken to collect the rhizosphere soil. The six rhizosphere soil samples were sieved (mesh size 0.5 mm) and combined into a single composite sample. Finally, the root system of each of the six plants per plot was combined into a composite sample and rinsed, and the fine roots (≤ 1 mm thick) were cut into 1- to 2-cm-long fragments. Three subsamples were collected from each pool of root fragments: two subsamples were transferred into two plastic Shandon™ tissue cassettes (Thermo Scientific™) for root colonization analysis and one subsample was stored in 1.5 ml tubes. Composite samples of bulk soil, rhizosphere soil, and roots were stored at −80°C.

### Root Colonization Analysis

The tissue cassettes containing the root fragments were stored in tap water acidified with a few drops of white vinegar at 4°C until all samples were processed. Root fragments were stained using the “ink and vinegar” technique (Vierheilig and Piché, 1998). Cassettes were boiled in 10% w/v KOH solution for 3 min, boiled in a 5% ink (Shaeffer black) and white vinegar solution for 3 min, soaked in acidified tap water for 20 min and stored in a 50% glycerol solution. Root samples were examined under a Zeiss Discovery V20 stereomicroscope coupled with an AxioCam ICC 5 camera (Carl Zeiss, Oberkochen, Germany). ZEN pro software v2012 (Carl Zeiss, Oberkochen, Germany) was used to digitize and visualize the root fragments. The percent of root length colonized was evaluated using the gridline intersection method (Giovannetti and Mosse, 1980). A total of 39,116 intersects were recorded. Colonization percentage was calculated as the ratio of colonized intersects divided by the total number of intersects and multiplied by 100.

### DNA Extraction

The UltraClean™ soil DNA Isolation Kit (MoBio, Laboratories, Carlsbad, CA) was initially used to isolate DNA from soil samples (48 out of 124). However, due to MoBio Laboratories not manufacturing anymore that kit during the wet lab stage of the study, the PowerSoil™ DNA Isolation Kit (Qiagen, Hilden, Germany) was then used to isolate DNA for the remaining soil samples and the rhizosphere samples. **Table S3** provides the list of the soil samples which were analysed with one or the other kit and the absence of difference between the AM fungal communities recovered with each kit is shown in supplementary material **Table S4**. The manufacturer’s instructions for both kits were followed, except that soil and rhizosphere DNA was eluted in 50 µl for the PowerSoil kit. DNA extractions from soil and rhizosphere samples were performed in duplicate and the duplicates were pooled.

Root fragments were put in 2-ml tubes containing Tungsten Carbide Beads of 3 mm, cooled in liquid nitrogen and placed immediately in a TissueLyser II instrument (Qiagen) for crushing mechanically the roots. A DNeasy® Plant Mini Kit (Qiagen, Valencia, CA) was used following the manufacturer’s instructions except that the final elution was done in 75 μl instead of 100 μl, and the flow-through from the first elution was reused for the second elution rather than using fresh elution buffer. The quantity and quality of the DNA extracts were first assessed on 1.5% agarose gel stained with GelRed^®^ (1:10000, Biotium, USA), run at 70 V for 60 min, and visualized using the Gel-Doc system (Bio-Rad Laboratories, Mississauga, ON). The quantity and quality of the DNA extracts were also assessed by means of Qubit Fluorometer 2.0 (Life Technologies, Burlington, ON, Canada), using the Qubit dsDNA HS assay kit. DNA extracts were stored at –20°C until use.

### DNA Amplification and Illumina Library Preparation

The AM fungal communities were characterized using the primer pair AML1/AML2 (Lee et al., 2008) which targets the V3-V4-V5 variable regions of the nuclear 18S small subunit (SSU) ribosomal RNA gene. The amplification was performed in 20 μl of reaction mix in triplicate as follows: 1 μl of gDNA, 200 μM of each dNTP, 2 mM of Mg^2+^, 0.8 μM of each primer, and 2.5 U of Q5 HighFidelity DNA Polymerase (NEBNext^®^ Q5 Hot Start HiFi PCR Master Mix). The thermocycling conditions were as follows: initial denaturation at 98°C for 30 s, 20 cycles at 98°C for 10 s, 64°C for 30 s, 65°C for 60 s, and final extension performed at 65°C for 5 min. The DNA was amplified in a Biometra TProfessional thermocycler (Biometra GmbH, Goettingen, Germany). The three amplicon replicates were pooled and purified using the QIAquick PCR Purification Kit (Qiagen, Valencia, CA) and eluted in 50 μl of elution buffer. This step is important to prevent interactions between the remaining primers during nested PCR. PCR products were visualized in a GelRed stained 1.5% agarose gel.

In order to comply with the sequencing length capacity of Illumina MiSeq^®^ Reagent Kit v3 (2 × 300 bp), a new primer pair yielding a 490-bp-length amplicon (including primers) was designed to target the V3-V4 region of the nuclear 18S rRNA gene: nu-SSU-0450-5′ (5′- CGCAAATTACCCAATCCC-3′) and nu-SSU-0899-3′ (5′-ATAAATCCAAGAATTTCACCTC-3′). Primers were named according to the primer nomenclature system of Gargas and DePriest (1996). The number in the primer name refers to the 5′ end position of the primer on the 18S sequence standard of *Saccharomyces cerevisiae* (GenBank accession Z75578). Primers were designed based on the guidelines provided by Integrated DNA Technologies (IDT Inc., San Diego, CA USA). Thermodynamic features of each primer are provided as supplementary material (**Table S5**). Purified PCR products amplified with AML1/AML2 were used as templates for nested PCR. A one to three bp “heterogeneity spacer” was introduced between the 3′ end of the adapter and the 5′ end of the primer pair nu-SSU-0450-5′/nu-SSU-0899-3′ to dampen the effect of the low sequence diversity issue of the MiSeq platform (**Table S6**, Fadrosh et al., 2014). The recipe for the amplification reaction was similar to the first-round PCR, except for the primer concentration which was 0.5 μM. The thermocycling conditions were as for the first round PCR except for the number of cycles which was reduced to 15 and the annealing temperature which was 59°C. The nested PCR was performed in triplicate and verified by electrophoresis on a GelRed-stained 1.5% agarose gel. Replicates were pooled.

Library preparation followed the protocol described in Stefani et al. (2020). Briefly, the PCR products from the nested PCR were purified using Agencourt AMPure^®^ XP beads (Beckman Coulter Inc., Indianapolis, IN, USA), normalized to 1 to 2 ng/μl with the SequalPrep™ Normalization Plate kit (ThermoFisher Scientific) and indexed using the Nextera index kit (Illumina, San Diego, CA, USA). Indexed amplicons were then purified and normalized. Purified indexed amplicons were quantified by qPCR using the LightCycler^®^ 480 system (Roche Molecular Systems Inc., Branchburg, NJ, USA) with the KAPA library quantification kit for Illumina platforms (KAPA Biosystems, MA, USA) in order to determine the volume of each sample to make up a 1-nM amplicon pool for library preparation.

Paired-end sequencing (2 × 300 bp) was carried out using the Illumina MiSeq^®^ sequencer for 500 cycles at the Molecular Technologies Laboratory of Agriculture and Agri-Food Canada Ottawa Research and Development Centre.

### Bioinformatic Analyses

The bioinformatic workflow is illustrated in **Figure S2** and its impact on the sequence dataset is described in the supplementary material. The raw demultiplexed sequences were processed in QIIME 2 v2020.2.0 (Bolyen et al., 2019). Paired-end sequences were denoised, dereplicated, and filtered for chimeras using the DADA2 plugin (Callahan et al., 2016), as implemented in QIIME 2. Sequences were trimmed in order to include only bases with quality scores > 35. The first 18 and 22 nucleotides of the 5′ end of the forward and reverse sequences, respectively, were trimmed. The 3′ end of the forward and reverse sequences was truncated at positions 266 and 261, respectively. Reads with number of expected errors higher than 1 were discarded. The number of sequences used to train the error model was set to 200,000. Amplicon sequence variants with a frequency of less than 0.1% of the mean sample depth were considered rare ASVs and removed. This threshold represents the MiSeq bleed-through between runs as reported by Illumina (https://github.com/LangilleLab/microbiome_helper/wiki/Amplicon-SOP-v2-(qiime2-2020.2)). De novo clustering using a threshold of 100% similarity was performed using *vsearch* (Rognes et al., 2016), as implemented in QIIME 2. This step was required because the use of degenerate fusion primers (**Table S6**) introduced one to three extra nucleotides in read length and the use of DADA2 (as implemented in QIIME 2) would have produced different ASVs for identical sequences of variable length. **Figure S3A** shows that the ASV richness in samples from each compartment was saturated with a sequencing depth of 5,000. **Figure S3B** showed that the number of analysed samples was appropriate to characterise the field resident AM fungal communities. Each sample was rarefied to 5,000 sequences which retained 1,680,000 (32.9%) sequences in 336 (90.3%) samples and 303 (95.6%) of the amplicon sequence variants (**Figure S4**). The taxonomic identification of each ASV was performed using a backbone phylogenetic tree as described in Stefani et al. (2020). The taxonomic assignment of each ASV is provided in **Table S7** and **Figure S5**.

### Alpha-Diversity Analyses

AM fungal diversity was estimated *via* the number of ASVs as a proxy of species richness. Venn diagrams were produced using the package *venndiagram* v1.6.20 (Chen and Boutros, 2011; Chen, 2018). The matrix used to calculate the relative abundance of the main AM fungal clades per block and per compartment was obtained using the rarefied ASV table. The ASV table was converted into a “biom” file, and the taxonomic information was added using the command *biom add-metadata*. Then the command *collapse_samples.py* (QIIME 1 v1.9.1) was used to combine repetitions from each block per microbiome. The barplots were produced using the R package *ggplot2* v3.3.0 (Wickham, 2016). The core AM fungal community was calculated on the rarefied datasets (raw abundance) using the function *core* and *plot_core* from R package *microbiome* v1.8.0. (Lahti and Shetty, 2019). Detection and prevalence thresholds were set to 0 and 50, respectively. Within-sample (alpha) diversity was calculated using the sample size- and coverage-based rarefaction and extrapolation (R/E) of the Hill numbers of species, i.e., richness (q = 0), Shannon diversity (q = 1, the exponential of Shannon entropy), and Simpson diversity (q = 2, the inverse of Simpson concentration), as implemented in the R package *iNEXT* version 2.0.19 (Chao et al., 2014; Hsieh et al., 2016). The Faith’s phylogenetic diversity index (Faith and Baker, 2006) was calculated using the QIIME 2 command *qiime diversity alpha-phylogenetic* on a RAxML phylogenetic tree that included 303 AM fungal ASVs as described in Stefani et al. (2020). Heatmaps showing the relative abundance of the core ASVs by cultivar for each compartment were produced using the R package *superheat* v0.1.0 (Barter and Yu, 2017). Principal component analysis was visualized using the function *fviz_pca_var* from the R package *factoextra* v1.0.7 (Kassambara and Mundt, 2020).

### Statistical Analyses

Linear models were used to investigate the effects of durum wheat cultivar and compartment (i.e. bulk soil, rhizosphere soil, and root) on the structure of AM fungal community. In order to analyse data sharing a similar distribution, variables (ASVs) with > 50% of non-zero values (i.e. core AM fungal community represented by 29 ASVs, hereafter identified as category ASVs_50+_), and variables with 10% to 50% of non-zero values (47 ASVs, category ASVs_10-50_) were analysed separately. Variables with less than 10% of non-zero values (227 ASVs, category ASVs_10-_) were ignored.

For the category ASVs_50+_, a principal component analysis (PCA) was realized on the correlation matrix with a varimax rotation. The function *PCA* from the R package *FactoMineR* v2.3, (Le et al., 2008) was used, with data scale to unite variance and nine principal components (74.6% of cumulative variance) retained based on the Kaiser criterion (i.e. with an eigenvalue higher than one). In order to avoid running the linear mixed effects analysis on 29 ASVs, a single ASV per principal component was selected with a saturation coefficient close to +1 or −1 (**Figure S6**). The linear mixed effects analysis was performed to investigate the relationship between the abundance of sequences and durum wheat cultivars (31), compartments (3) and ASVs (9). Cultivars, compartments, and ASVs (with interaction terms) were set as fixed factors and block, block × cultivar, and block × cultivar × compartment were set as random factors. A squared root transformation was performed on the outcome value to respect the assumptions of the model. Means are presented on the original scale, while the *p* values come from the model on transformed data. For the category ASVs_10-50_, ASVs were dichotomized as null or non-null values. Principal component analysis was realized on the tetrachoric correlation matrix. Three dimensions (58.9% of cumulative variance) were retained based on variance criteria (one dimension should explain > 10% variance), with eigenvalues ranking from 0.83 to 0.25. Again, a single ASV showing the highest positive or negative saturation coefficient was selected per dimension, leading to the selection of ASV021, ASV030 and ASV031. A generalized linear mixed model (GLMM), with a logit link, was then performed using the same technique as for the LMM. Finally, linear mixed models were also used to investigate the effects of cultivars and compartments on diversity indices (ASV richness, Shannon and Simpson diversity, Faith’s phylogenetic diversity index), with random effects for block and block × cultivar. Heterogeneous variances were modelled for Shannon diversity values. Linear mixed models were also used to test the effects of cultivars on the colonisation rate, with a random effect for block. In all LMM and GLMM, multiple comparisons using Tukey adjustment were done for significant effects.

Statistical analyses were done using R v3.6.3 (R Core Team, 2020), and the following packages: *car* *v3.0-7* (Fox and Weisberg, 2019), *emmeans v1.4.6* (Lenth, 2020), *factoextra v1.0.7* (Kassambara and Mundt, 2020), *MASS v7.3-51.5* (Venables and Ripley, 2002), *mgcv v1.8-31* (Wood, 2004), *moments v0.14* (Komsta and Novomestky, 2015), *nlme v3.1-144* (Pinheiro et al., 2020), *psych v1.9.12.31* (Revelle, 2020), *reshape v0.8.8* (Wickham, 2007), and *sjmisc v2.8.4* (Lüdecke, 2018).

## Results

### Characterization of the AM Fungal Community Associated With Durum Wheat

**Figure 1:**
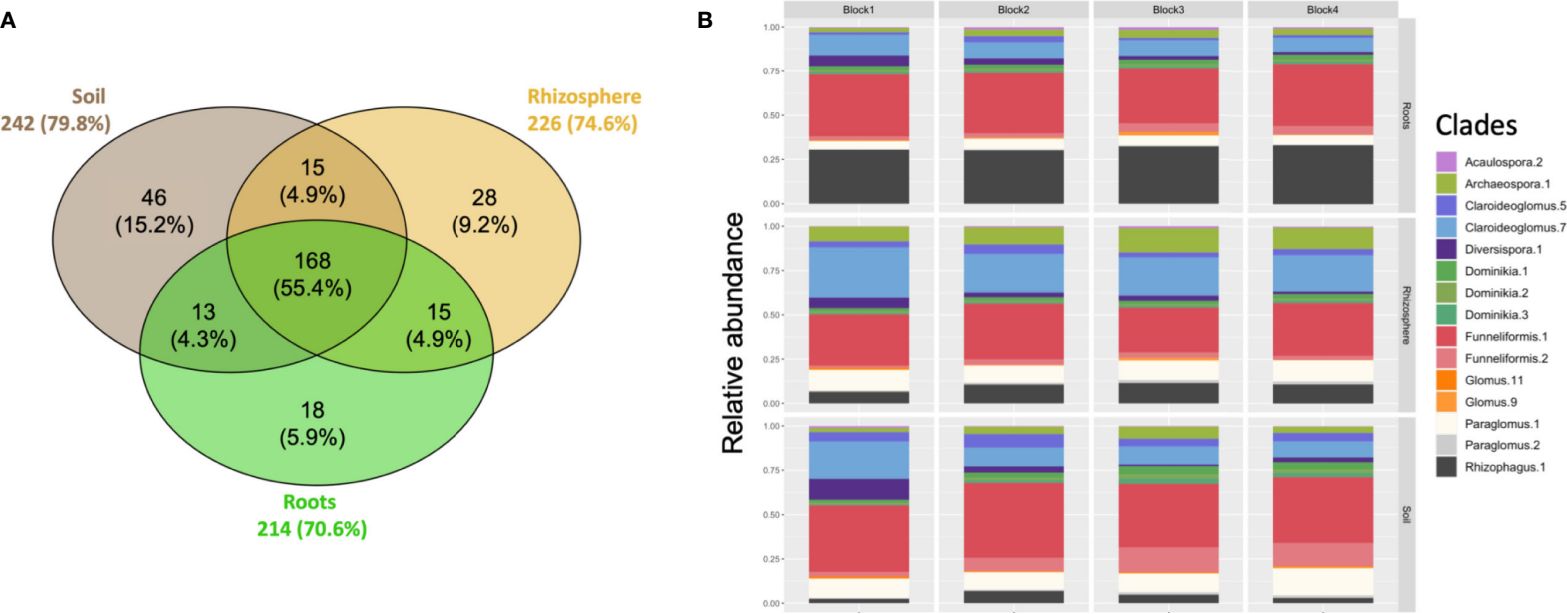
**(A)** Venn diagram showing overlap of amplicon sequence variants (ASVs) between soil, rhizosphere, and root compartments. The size of the circles is proportional to the number of ASVs recorded in each compartment. Each sample was randomly subsampled to a common sequencing depth of 5000 sequences. **(B)** Taxonomic profile of AM fungi recovered in soil, rhizosphere and root compartments for each block. The clades with a relative abundance < 1% are not shown.

A total of 303 ASVs belonging to Glomeromycota were recovered in the rarefied dataset. About 50% of the 303 ASVs were shared between the bulk soil, rhizosphere soil, and root samples (**Figure 1A**). The number of ASVs recorded was relatively similar between soil (242 ASVs), rhizosphere (226 ASVs) and root (214 ASVs) compartments. The AM fungal community was dominated by the genera *Funneliformis, Claroideoglomus, Paraglomus*, and *Rhizophagus* (**Figure 1B**). Sequences from the genus *Funneliformis* were the most abundant (19 ASVs), with a relative abundance ranging from 30% in roots to 45% in soil. ASV001 was the most abundant (21.6% of sequences) and the sequences were homologous to the 18S sequence of *F. mosseae* BEG12. The clade Claroideoglomus-7 was the second most abundant in bulk soil (12%) and rhizosphere soil (22%). It included 9 ASVs with sequence similarities close to the species *C. claroideum, C. etunicatum, C. lamellosum*, and *C. luteum* (**Figure S5**). The clade Paraglomus-1 (8 ASVs) was the third most abundant in bulk soil (11%) and rhizosphere soil (11%). A BLAST search for these 8 ASVs showed that their sequences were closely related to 18S sequences assigned to *P. occultum* (MN793990) and *P. laccatum* (MN517120). However, these ASVs did not cluster with the 18S sequences from well identified herbarium cultures of *P. occultum* (HA771 and IA702, **Figure S5**). The clade Archaeospora-1 (14 ASVs) was well represented in rhizosphere soil with an average relative abundance of 10%. It is interesting to observe the increasing abundance of the clade Rhizophagus-1 (21 ASVs) from bulk soil (4.1%), to rhizosphere soil (9.5%), to roots (30.6%). The clade Rhizophagus-1 included sequences of well-identified herbarium cultures of species *R. irregularis*, *R. vesiculifer*, and *R. fasciculatum* (**Figure S5**).

**Figure 2:**
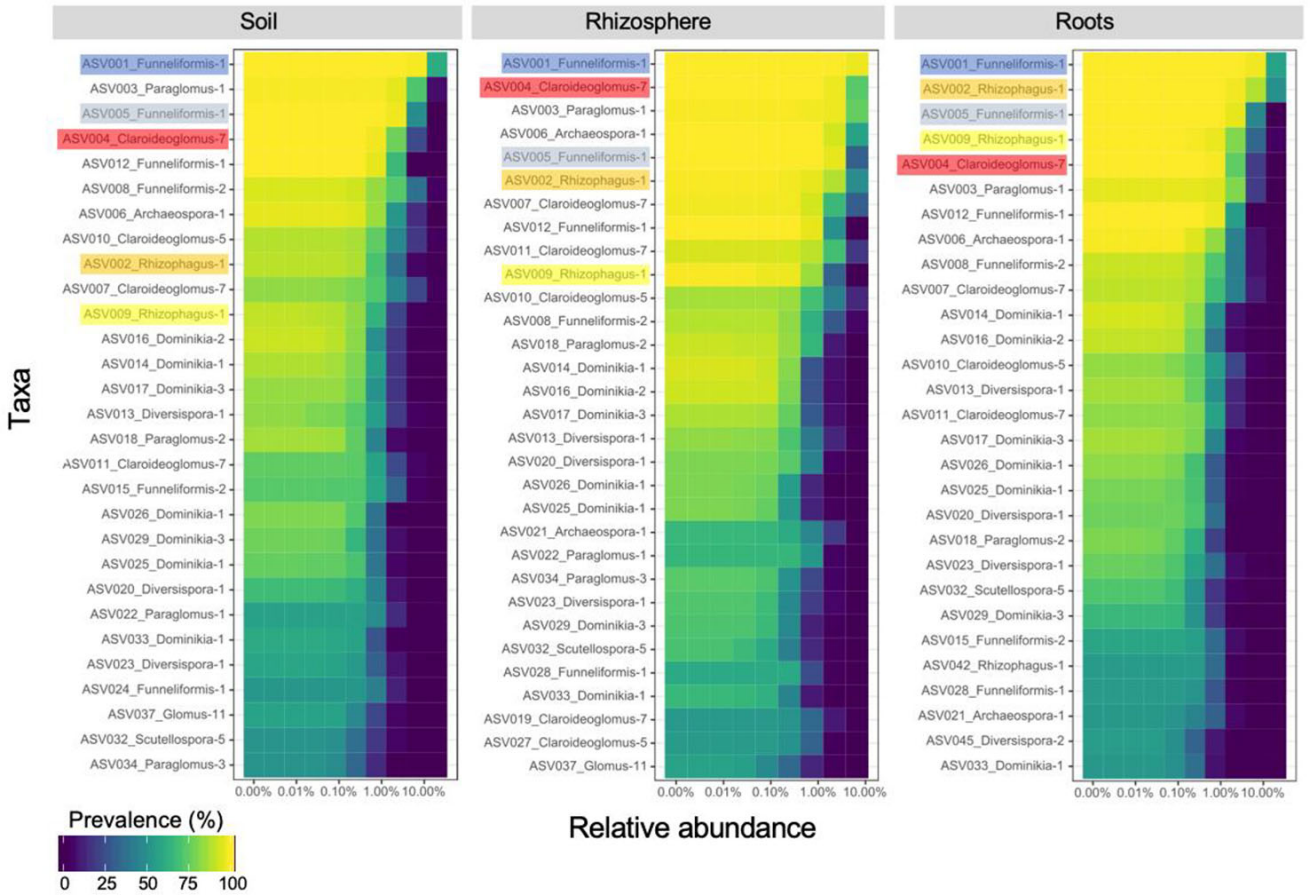
Core community of arbuscular mycorrhizal fungi recovered in soil (29 ASVs), rhizosphere (30 ASVs) and root (29 ASVs) samples. The top five amplicon sequence variants recovered in root samples are highlighted to emphasize their ranking in rhizosphere and soil samples. ASVs are ranked on the ordinate axis from the most (top) to the least (bottom) prevalent in each compartment while they are ordered by ascending relative abundance on the abscissa axis.

The core AM fungal community (i.e. ASVs present in > 50% of the samples) included 29, 30 and 29 ASVs for the bulk soil, rhizosphere soil, and root compartments, respectively (**Figure 2**) and 29 ASVs (category ASVs_50+_) when the three compartments were considered together. These ASVs were assigned to 8 genera (*Archaeospora, Claroideoglomus, Diversispora, Dominikia, Funneliformis, Glomus, Paraglomus, Rhizophagus*), and represented five families (Archaeosporaceae, Claroideoglomeraceae, Diversisporaceae, Glomeraceae, Paraglomeraceae), four orders (Archaeosporales, Diversisporales, Glomerales, Paraglomerales) and three classes (Archaeosporomycetes, Glomeromycetes, and Paraglomeromycetes).

### Cultivar and Compartments Effects on AM Fungal Community

The lowest percentages of root colonisation were recorded in Arnautka (57.8% ± 14.8), Hercules (59.8% ± 6.7) and AC Pathfinder (62.8% ± 10.4) while the highest percentages were observed in roots from Transcend (77.3% ± 19), Enterprise (79.8% ± 7.3) and AC Navigator (84% ± 17.5, **Figure S7**). However, the effect of cultivars on the percentages of root colonisation was not significant (*F*_30,90_ = 1.02, *p* = 0.45).

**Figure 3:**
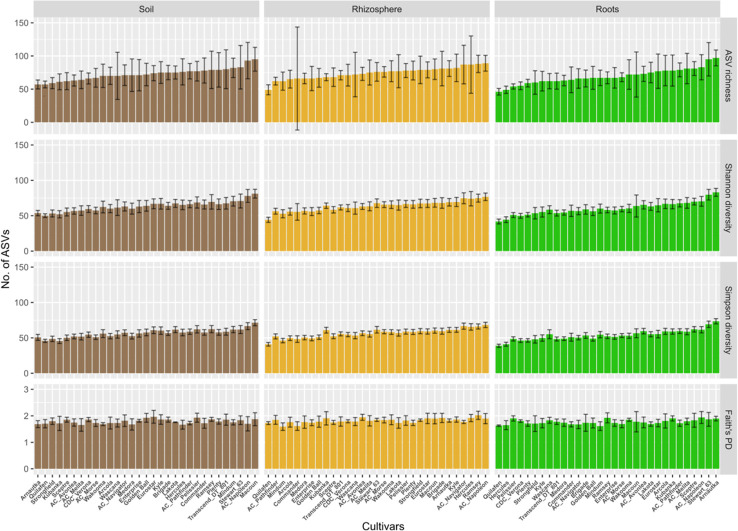
Diversity measured in soil, rhizosphere and root samples for each of the five landraces and 26 durum wheat cultivars. The ordinate displays Hill numbers of three orders: ASV richness (q = 0), Shannon diversity (q = 1), and Simpson diversity (q = 2), and Faith’s Phylogenetic diversity. Cultivars are sorted according to the mean ASV richness observed in root samples, from the lowest to the highest. Error bars represent estimated bootstrap standard error.

The average ASV richness per cultivar and compartment ranged between 50 and 100 (**Figure 3**). Only cultivar Quilafen consistently showed low average ASV richness for each compartment (57, 49 and 46 for bulk soil, rhizosphere soil, and roots, respectively). No clear relationship in the average ASVs richness between compartment was observed for the other cultivars. For example, 57 ASVs were recorded in bulk soil samples under Arnautka while 97 ASVs were recorded in its roots. High number of ASVs in root samples does not mean high percentage of root colonisation because the percentage of root colonization was lowest in Arnautka (57.8% ± 14.8). The opposite situation was observed in Quilafen which had the lowest average number of ASVs in roots, and the fifth highest average rate of root colonization (76% ± 7.8).

No significant effect of cultivars (*F*_30,90_ = 1.23, *p* > 0.05) and compartments (*F*_2,150_ = 1.90, *p* > 0.05) was observed on ASV richness (**Figure 3**). No significant effect of cultivars (*F*_30,90_ = 1.15, *p* > 0.05) and compartments (*F*_2,150_ = 2.38, *p* > 0.05) was observed on Faith’s phylogenetic diversity index. A compartment effect was found significant on Shannon (*F*_2,150_ = 17.7, *p* < 0.0001) and Simpson diversity (*F*_2,150_ = 34.2, *p* < 0.0001). The Tukey *post hoc* tests showed that estimated marginal means of both indices were significantly lower (*p* < 0.0001) in roots than in bulk soil and rhizosphere soil samples (data not shown), which indicates a degree of dominance in the AM fungal community recorded in the root samples.

**Figure 4:**
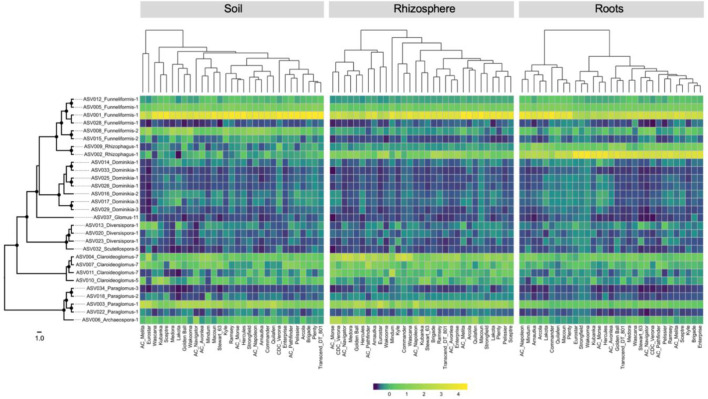
Heatmaps showing the relative abundance of each of the 29-core amplicon sequence variants recorded in soil, rhizosphere, and root compartments for the five landraces and 26 durum wheat cultivars. Left: RAxML phylogeny of 29 core ASVs, black circles on the nodes represent bootstrap values > 70. The scale represents the branch length corresponding to expected substitutions per site. Top: cladogram showing the relationships between each cultivar based on the distance matrix calculated on ASV relative abundance.

The heatmaps of the relative abundance of the 29 core ASVs by compartment and cultivar did not show major differences between cultivars for most of the ASVs (**Figure 4**). A clear shift in the relative abundance of ASV001 (Funneliformis-1) was visible in each compartment but it did not involve the same groups of cultivars, with exception of Eurostar. The heatmap based on the root compartment clearly showed an antagonist behavior between ASV001 and ASV002 (Rhizophagus-1) for two clusters of 9 and 22 cultivars. The dendrograms of core ASVs community profiles were statistically not similar, according to the Bray-Curtis distance (**Table S8**).

**Figure 5:**
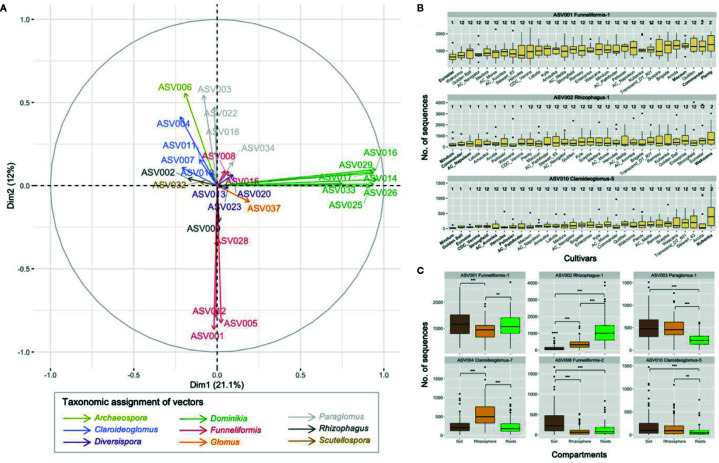
**(A)** Principal component analyses on 29 ASVs with non-zero values in > 50% of the samples across the three compartments (category ASV_50+_). The first two principal components explained 33.1% of variance. **(B)** Boxplots showing the abundance of the three ASVs for which significant differences were recorded between the pairwise comparisons of the 31 cultivars of durum wheat. Boxplots with a different number indicate significant differences between cultivars (*p* < 0.05). Boxplots were ordered by increasing mean abundance. **(C)** Boxplots showing the abundance of the six ASVs for which the significant differences were found between soil, rhizosphere and root compartments. Non-significant differences are not shown, ** and *** indicate *p* values < 0.01 and < 0.001, respectively.

The PCA performed on the 29 core ASVs (category ASVs_50+_) showed that ASVs from the genera *Dominikia* and *Funneliformis* were highly correlated with the first and second principal component, respectively (**Figures 5A** and **S6**). ASVs from the genera *Rhizophagus* (ASV002), *Claroideoglomus* (ASV010), *Scutellospora* (ASV32) and *Paraglomus* (ASV003) were correlated with the fourth, sixth, seventh, and eighth principal component, respectively (**Figure S6**). The linear mixed model analysis performed on one representative ASV per principal component showed that the two-way interactions cultivar × ASV (*F*_240,1944_ = 1.4, *p* = 0.0003) and compartment × ASV (*F*_16,1944_ = 71.4, *p* < 0.0001) were significant. The pairwise comparisons of estimated marginal means between cultivars were significantly different (*p* < 0.05) for ASV001 (Funneliformis-1), ASV002 (Rhizophagus-1), and ASV010 (Claroideoglomus-5), following Tukey’s correction. For ASV001, the estimated marginal mean calculated for Eurostar was significantly inferior to Commander, Macoun, and Plenty (**Figure 5B**). The same differences between these cultivars are expected for ASV005 and ASV012 since they are negatively correlated on principal component 2 (r = −0.925 and r = −0.917, respectively), similarly to ASV001 (r = −0.925, **Figure S6**). For ASV002, the estimated marginal means calculated for AC Napoleon, Lakota, and Mindum were significantly inferior to Wakooma. For ASV010, the estimated marginal means calculated for AC Avonlea, AC Pathfinder, CDC Verona, Eurostar, Golden Ball, Hercules, Macoun, Mindum, Strongfield were significantly inferior to cultivar Kubanka. Finally, the abundance recorded for six out of nine ASVs were significantly different between the compartments (**Figure 5C**). Only ASV002 (Rhizophagus-1) was significantly more abundant in roots than in rhizosphere and bulk soil. ASV003 (Paraglomus-1), ASV008 (Funneliformis-2), and ASV010 (Claroideoglomus-5) followed the opposite dynamic since their abundance were significantly lower in roots compared to rhizosphere and/or soil. ASV001 (Funnliformis-1) was significantly less abundant in rhizosphere compared to soil and roots while ASV004 (Claroideoglomus-7) showed the opposite pattern. Finally, the generalized linear mixed model analysis performed on the category ASVs_10-50_ showed no significant difference (data not shown).

## Discussion

### AM Fungal Communities Associated With Durum Wheat

Results from this study provide an in-depth overview of the AM fungal communities associating with wheat genotypes representative of Canadian durum, in a humid climate. The core AM fungal community associated with durum wheat recorded in Eastern Canada was relatively similar to that recorded in the Canadian prairie (Ellouze et al., 2018). Results from the current study and from Ellouze et al. (2018) showed that the genus *Funneliformis* was core for durum wheat. In the current study, a sequence homologous to that from the culture of *Funneliformis mosseae* BEG12 was the most abundant in soil, rhizosphere, and root compartments. In Ellouze et al. (2018), *Funneliformis* (50.3% of the reads), *Claroideoglomus* (10.7%) and *Dominikia* (2.9%) were the main genera recorded in soil and root samples associated with five landraces and 32 durum wheat cultivars grown on the Brown and Black soils in the Canadian prairie. In the current study, the genera *Claroideoglomus* and *Paraglomus* were also part of the five most abundant genera recorded in each compartment. The genus *Dominikia* was also included in the core AM fungal community with a relative abundance ranging from 4% to 6% across the three sampled compartments. A sequence from an unknown *Archaeospora* species (98.5% of pairwise similarity with the 18S sequence of the herbarium culture of *A. trappei* NB112) was part of the 10 most abundant sequences in bulk soil, rhizosphere soil, as well as in roots. Ellouze et al. (2018) reported the genus *Archaeospora* was abundant in root samples from the Brown soil site only. None of the ASVs were assigned to the genus *Gigaspora*. It has been previously observed that taxa from *Gigaspora* and *Scutellospora* tended to disappear in agricultural field under conventional management such as tillage (Hamel et al., 1994; Boddington and Dodd, 2000; Kabir, 2005). Here the experimental field was tilled in fall 2015 and harrowed in spring 2016. A total of 317 ASVs was recorded in the current study while 190 AM fungal OTUs were associated with durum wheat in the Prairies using a similarity threshold of 97% to cluster 18S AM fungal sequences. As discussed in Stefani et al. (2020) and in Schlaeppi et al. (2016), 18S sequences from different AM fungal species are lumped together at a similarity threshold of 99%, making the ASVs approach the least inaccurate as a proxy for species for datasets based on the 18S sequences of the nuclear ribosomal DNA. However, using ASVs based on 18S sequences as a proxy for AM fungal species could overestimate species richness. For instance, within the clade Rhizophagus-1, 10 ASVs were closely related to the sequences from cultures of *R. irregularis* (DAOM 197198, MUCL 43195, and W4533). The pairwise similarities between these 10 ASVs ranged from 98.9% (5 diverging nucleotides) to 99.8% (1 diverging nucleotide). Maeda et al. (2018) determined that the genome of *R. irregularis* DAOM 181602 was characterized by ten rDNA paralogs which the mean intra-genomic similarity was 99.91% (SD = 0.06) for the 18S gene. Therefore, it is possible that 490 bp long 18S fragments diverging by a single nucleotide could represent paralogs from the same species. Similarly, ten ASVs closely related to the sequences from well characterised cultures of *F. mosseae* (BEG12, FL126, DAOM 212595) had pairwise similarities ranging from 99.6% to 99.8%, i.e. two to one nucleotide of divergence, respectively. With exception to *R. irregularis*, the number of rDNA paralogs and their intra-genomic variability remain unknown in the other species of AM fungi. While it is undetermined if a unique similarity threshold can be applied across the phylum Glomeromycota to accurately recognise species, a threshold ranging from 99.5% to 100% seems to be safe for not lumping into the same contig 18S sequences from closely related species.

ASV002 (100% of pairwise similarity with *R. irregularis* DAOM 181602/DAOM 197198) was the second most abundant sequence recorded in the root samples, while it was ranked in 11^th^ position in bulk soil. Its relative abundance in roots was significantly more important than in rhizosphere and bulk soil compartments. This clearly shows the preferential selection of the host plant for *R. irregularis*. Compared to *Glomus custos* and *G. aggregatum*, Kiers et al. (2011) showed that *R. irregularis* (identified in the publication as *G. intraradices*) was the most cooperative species as it provided to its host the best rate of nutrient exchange (more phosphorus for less carbon), resulting in a host preference for resources allocation to *R. irregularis*. This makes *R. irregularis* a strong competitor in *in vivo* or *in vitro* system (Engelmoer et al., 2014), but also under field conditions as suggested by the data from the current study. Among the five most abundant sequences recorded in roots, two were from the genus *Rhizophagus* and two from the genus *Funneliformis*.

ASVs from all four AM fungal orders were recovered, showing that the nested PCR approach based on the primer set AML1/AML2 and the new primer set nu-SSU-0450-5′/nu-SSU-0899-3′ is able to target all AM fungal taxa. Moreover, non-AM fungal sequences represented only 6.1% of the sequences obtained after the quality filtering. Berruti et al. (2017) used a similar approach with a nested PCR based on the primer set AML1/AML2 for the first round PCR and the primer set AMADf/AMDGR (Sato et al., 2005) for the second round PCR to characterize the AM fungal community in roots and soils of three mountain vineyards. Both primer sets nu-SSU-0450-5′/nu-SSU-0899-3′ and AMADf/AMDGR target the V3-V4 regions of the 18S and are able to recover all known AM fungal lineages when used in combination with the AM fungi specific primer set AML1/AML2 (Lee et al., 2008). The new primer set nu-SSU-0450-5′/nu-SSU-0899-3′ amplifies a fragment slightly longer (490 bp) than the primer set AMADf/AMDGR (423 bp). This length is compatible with 2 × 300 paired-end sequencing and allows a 37 bp of overlap between forward and reverse reads once they were truncated in 3′ position due to decrease in quality.

### Cultivar Impact on AM Fungal Communities

To our knowledge, results from this study provide the most comprehensive characterisation of the AM fungal communities associated with durum wheat under field conditions. However, our results were obtained in one field trial using a non-restricted pool of durum wheat and at the time of sampling, that may have filtered the community in bulk soils, rhizosphere soils, and roots. A total of 317 ASVs were recorded representing the four AM fungal orders, thus a filtering effect on the AMF pool due to a single site and sampling time is unlikely. The levels of AM fungal diversity were similar between each cultivar, in all three compartments examined. However, the results clearly show a differential affinity of some cultivars for ASVs related to *F. mosseae* (ASV001, ASV005, and ASV010), *R. irregularis* (ASV002), and *Claroideoglomus* sp. (ASV010). ASV001 and ASV002 were the most abundant in the whole dataset and were assigned to genera previously identified as predominant in the AM fungal community associated with durum wheat growing in the dry environment of the Canadian prairie (Ellouze et al., 2018). Cultivars with strong affinity for *F. mosseae* had less affinity for *R. irregularis* and vice and versa. Indeed, the abundance of ASV001 was the lowest in cultivars Eurostar, Wakooma, and Golden Ball while the abundance of ASV002 was among the highest for these cultivars. Moreover, the responsiveness of cultivars Eurostar and Golden Ball to ASV010 (*Claroideoglomus* sp.) was limited compared to the other cultivars. Overall results showed that the genotypic differences between the five landraces and 26 durum wheat cultivars had only a minor impact on the structure of the AM fungal community. This suggests that the symbiotic signalling system (Bonfante and Requena, 2011) and the molecules (i.e. flavonoids, strigolactones, Steinkellner et al., 2007) released by the durum wheat cultivars to initiate the mutualistic interaction with AM fungi are well conserved for each genotype and that the set of genes involved with the recognition of the Myc-factors (pre-physical contact stage) and with the establishment of the mycorrhization (post-physical contact stage) were only marginally altered through the breeding. Tian et al. (2019) showed that 2360 genes were differentially expressed in the roots of *Triticum aestivum* under the influence of the molecular signals produced by *R. irregularis*. Zhao et al. (2014) showed that the orchid mycorrhizae trigger in the host the induction of various genes involved with cell wall modification or defence-related phytohormone and phosphate transport. It is possible that the genotypic differences between durum wheat cultivars lead to slightly different molecular interactions with some AM taxa. This could result in less compatible partners featured by a less abundant fungal biomass, leading to less sequence count.

Despite Arnautka, Hercules, and AC Pathfinder cultivars were less colonized than Transcend, Enterprise, and AC Navigators ones, the analysis of the percentages of root colonisation showed non-significant variation within durum wheat cultivars. However, a spread of 26% between the cultivars showing the lowest and highest percentages of colonisation was observed. Because the phenotypic variation of these cultivars has not been characterized so far, one cannot exclude that some genotypes have had variable phenotypical traits (such as root branching, biomass, shoot branching) that could result in different level of root colonization. The non-significant differences observed in the percentages of root colonisation between cultivars grown under field conditions contrasts with experiments performed in greenhouse with commercial inoculum of *R. irregularis* DAOM 197198 (Singh et al., 2012; Ellouze et al., 2016). Singh et al. (2012) inoculated five cultivars of durum wheat under low and medium fertility conditions. The type of cultivar was identified as having a significant effect on the percentages of root colonization at both low and medium soil fertility, and the cultivars showing percentages of root colonisation significantly lower or higher varied according to the levels of fertility. At low and medium fertility, the cultivar “Commander” had the highest and lowest percentages of colonisation respectively. The same trend was observed with “Commander” and “Pathfinder” in Ellouze et al. (2016). In the current study, the cultivar “Commander” had the fifth lowest average colonisation percentage. In a pot trial carried out in greenhouse and involving a set of 94 bread wheat genotypes, Lehnert et al. (2017) reported signiﬁcant genotypic differences with regard to root colonization with a blend of three AM species (*Rhizophagus intraradices, Claroideoglomus claroideum*, and *C. etunicatum*). The authors also identified 30 signiﬁcant markers (representing six quantitative trait loci (QTL) regions) associated with root colonization, and they estimated that the heritability for root colonization was moderate. This suggests it is possible to improve root colonization by breeding. Similarly, De Vita et al. (2018) investigated the percentages of root colonisation and its genetic basis in the plant host by inoculating 108 durum wheat cultivars with *F. mosseae* and *R. irregularis*. They identified seven putative QTL associated with mycorrhizal susceptibility with each AM species and reported high variability in the percentage of root colonisation. These results suggest a complex genetic control of root colonisation.

Surprisingly, the percentages of root colonisation were very different between greenhouse experiments (Singh et al., 2012; Ellouze et al., 2016; De Vita et al., 2018) and the current field-based study while the same approach based on gridline intersect method (Giovannetti and Mosse, 1980) was used. Indeed, the average colonisation percentage recorded across all the cultivars was high (71%), with a spread of 26% between the cultivars showing the lowest (Arnautka) and highest (AC Navigator) percentages of colonisation. The percentages of root colonisation recorded in Singh et al. (2012); Ellouze et al. (2016) and De Vita et al. (2018) ranged between 5% and 45%. In their study on the effect of domestication on AM association at different fertility regimes, Martín-Robles et al. (2017) found better AM symbiotic development at low P fertility levels, in both domesticated crops and wild progenitors. However, P fertility was limited either in the greenhouse experiments as in the current field study. High percentages of root colonization here likely reflect other field conditions conducive to AM symbiotic development. The colonization percentages reported here are in line with what Graham and Abbott (2000) observed from “aggressive colonizers” at low P fertility (50–89% of root length colonization). In their case the aggressive colonizers included species such as *Scutellospora calospora, Glomus invermaium, Acaulospora laevis*, and *Gigaspora decipiens* inoculated onto specimens of Kulin wheat. These species triggered growth depression and reduced sucrose concentration in roots.

## Conclusion

Using deep 18S rDNA sequencing, the AM communities associating with the historical set of durum wheat genotypes in the field under an humid climate were comprehensively characterised and allowed to detect minor impacts of the cultivars on the structure of the AM fungal community. The hypothesis that different cultivars host distinct AM fungal communities is not supported in durum wheat, contrary to what some previous studies using other plant species have suggested. The genetic variation among durum wheat genotypes seems to be too narrow to select for specific plant-AM fungal associations from field resident AM fungal communities, using traditional breeding techniques. However, few cultivars had a differential responsiveness to *F. mosseae*, *R. irregularis*, and *Claroideoglomus* sp. Because the field trial was performed in a humid climate in Eastern Canada, results were not influenced by variation in soil moisture. This field trial along with the ones performed in the Canadian prairie examined the AM associations formed between durum wheat genotypes and resident AM fungi. In these three ecoregions, *F. mosseae* and *R. irregularis* were the main taxa recruited by durum wheat.

## Data Availability Statement

The Illumina data generated in this study were deposited in the NCBI Sequence Read Archive and are available under project number PRJNA645613.

## Author Contributions

CH designed the project and supervised field work. RK and YR designed the project and provided the biological material. SD performed the sampling, the DNA isolation and estimated the root length colonization. FS designed the primers nu-ssu-0450-5′/nu-ssu-0899-3′. FS and MH supervised laboratory work. FS and ML analyzed the data. ML performed data visualization. FS wrote the paper. MH and CH edited and provided critical review of the manuscript. All authors contributed to the article and approved the submitted version.

## Funding

This work was supported by funding from the NSERC Discovery grant (RGPIN-2018-04178, MH) and from the Agriculture and Agri-Food Canada through the projects J-000617 (CH) and J-002272 (FS).

## Conflict of Interest

The authors declare that the research was conducted in the absence of any commercial or financial relationships that could be construed as a potential conflict of interest.
