# Supplementary material for: Similar Arbuscular Mycorrhizal Fungal Communities in 31 Durum Wheat Cultivars (Triticum turgidum L. var. durum) Under Field Conditions in Eastern Canada
Source: Front Plant Sci. 2020 Aug 11;11:1206. doi: 10.3389/fpls.2020.01206 (PMC7431883; doi:10.3389/fpls.2020.01206)
Supplement: Supplementary file 1 [file DataSheet_1.pdf]

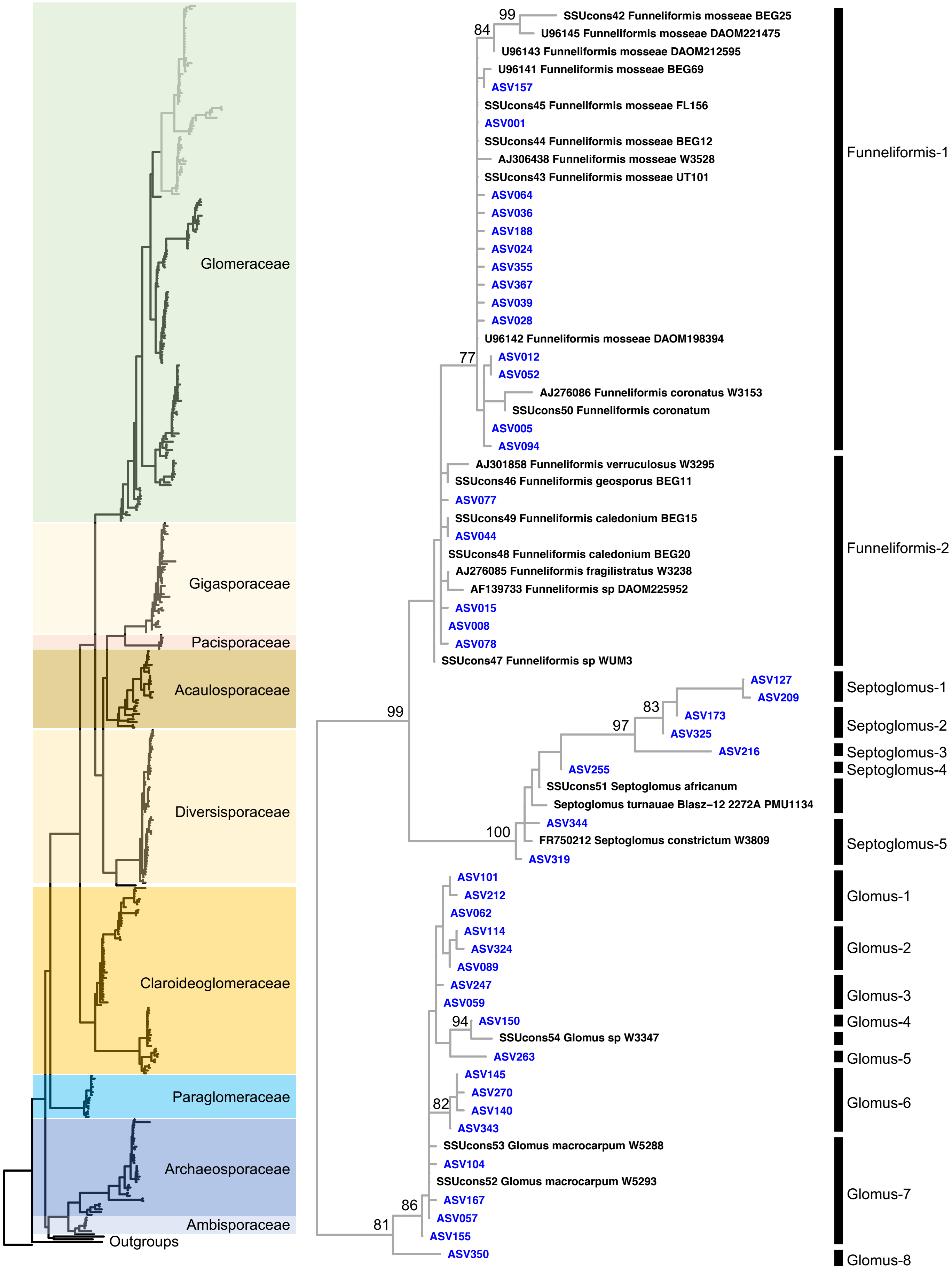

Glomeraceae
Gigasporaceae
Pacisporaceae
Acaulosporaceae
Diversisporaceae
Claroideoglomeraceae
Paraglomeraceae
Archaeosporaceae
Ambisporaceae
Outgroups
SSUcons42 Funneliformis mosseae BEG25
U96145 Funneliformis mosseae DAOM221475
U96143 Funneliformis mosseae DAOM212595
U96141 Funneliformis mosseae BEG69
ASV157
SSUcons45 Funneliformis mosseae FL156
ASV001
SSUcons44 Funneliformis mosseae BEG12
AJ306438 Funneliformis mosseae W3528
SSUcons43 Funneliformis mosseae UT101
ASV064
ASV036
ASV188
ASV024
ASV355
ASV367
ASV039
ASV028
U96142 Funneliformis mosseae DAOM198394
ASV012
ASV052
AJ276086 Funneliformis coronatus W3153
SSUcons50 Funneliformis coronatum
ASV005
ASV094
AJ301858 Funneliformis verruculosus W3295
SSUcons46 Funneliformis geosporus BEG11
ASV077
SSUcons49 Funneliformis caledonium BEG15
ASV044
SSUcons48 Funneliformis caledonium BEG20
AJ276085 Funneliformis fragilistratus W3238
AF139733 Funneliformis sp DAOM225952
ASV015
ASV008
ASV078
SSUcons47 Funneliformis sp WUM3
ASV127
ASV209
ASV173
ASV325
ASV216
ASV255
SSUcons51 Septoglomus africanum
Septoglomus turnauae Blasz–12 2272A PMU1134
ASV344
FR750212 Septoglomus constrictum W3809
ASV319
ASV101
ASV212
ASV062
ASV114
ASV324
ASV089
ASV247
ASV059
ASV150
SSUcons54 Glomus sp W3347
ASV263
ASV145
ASV270
ASV140
ASV343
SSUcons53 Glomus macrocarpum W5288
ASV104
SSUcons52 Glomus macrocarpum W5293
ASV167
ASV057
ASV155
ASV350
99
84
77
99
83
97
100
94
82
86
81
Funneliformis-1
Funneliformis-2
Septoglomus-1
Septoglomus-2
Septoglomus-3
Septoglomus-4
Septoglomus-5
Glomus-1
Glomus-2
Glomus-3
Glomus-4
Glomus-5
Glomus-6
Glomus-7
Glomus-8

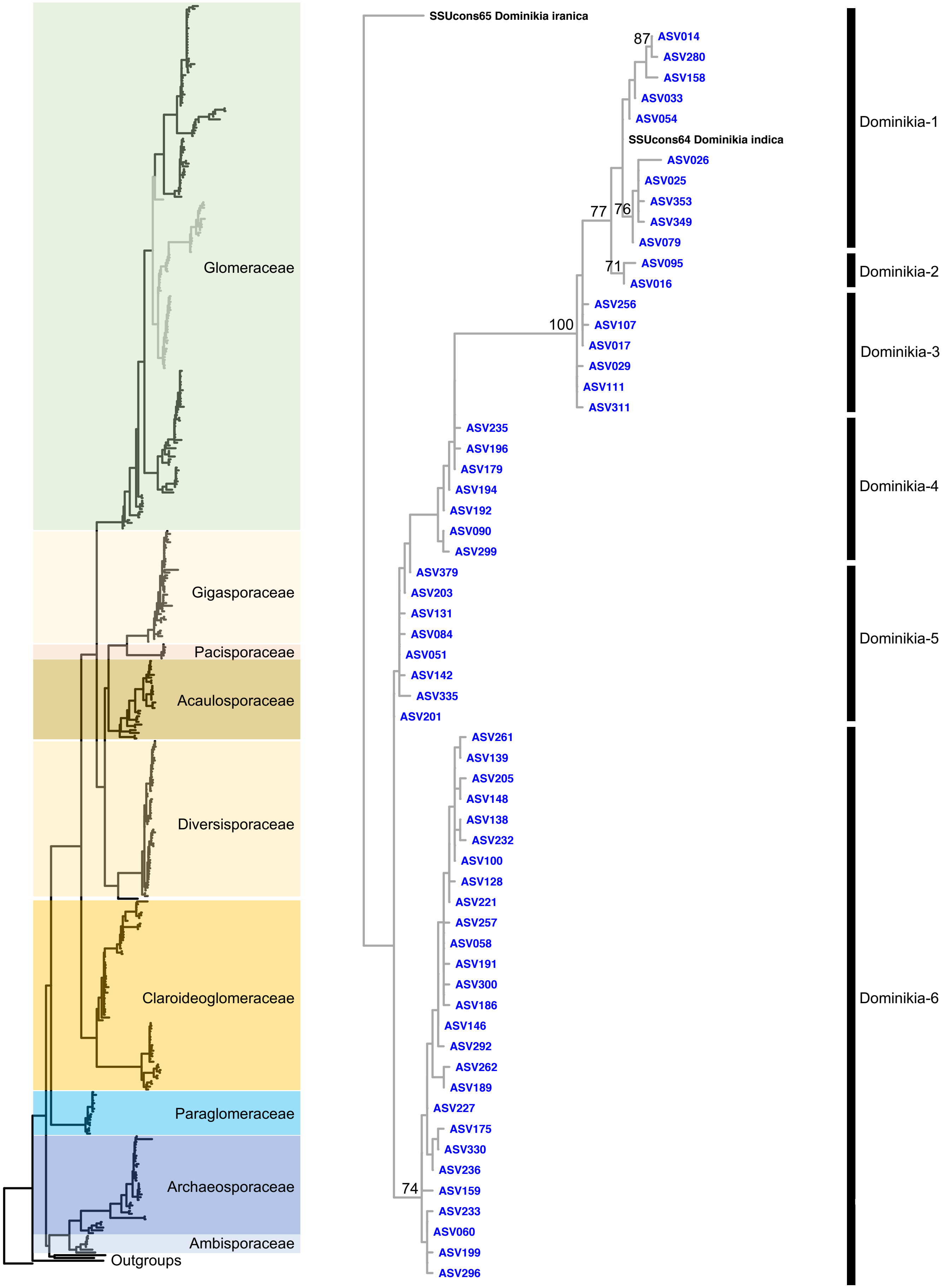
Glomeraceae
Gigasporaceae
Pacisporaceae
Acaulosporaceae
Diversisporaceae
Claroideoglomeraceae
Paraglomeraceae
Archaeosporaceae
Ambisporaceae
Outgroups
SSUcons65 Dominikia iranica
87
ASV014
ASV280
ASV158
ASV033
ASV054
SSUcons64 Dominikia indica
ASV026
ASV025
77
76
ASV353
ASV349
ASV079
71
ASV095
ASV016
ASV256
100
ASV107
ASV017
ASV029
ASV111
ASV311
ASV235
ASV196
ASV179
ASV194
ASV192
ASV090
ASV299
ASV379
ASV203
ASV131
ASV084
ASV051
ASV142
ASV335
ASV201
ASV261
ASV139
ASV205
ASV148
ASV138
ASV232
ASV100
ASV128
ASV221
ASV257
ASV058
ASV191
ASV300
ASV186
ASV146
ASV292
ASV262
ASV189
ASV227
ASV175
ASV330
ASV236
74
ASV159
ASV233
ASV060
ASV199
ASV296
Dominikia-1
Dominikia-2
Dominikia-3
Dominikia-4
Dominikia-5
Dominikia-6

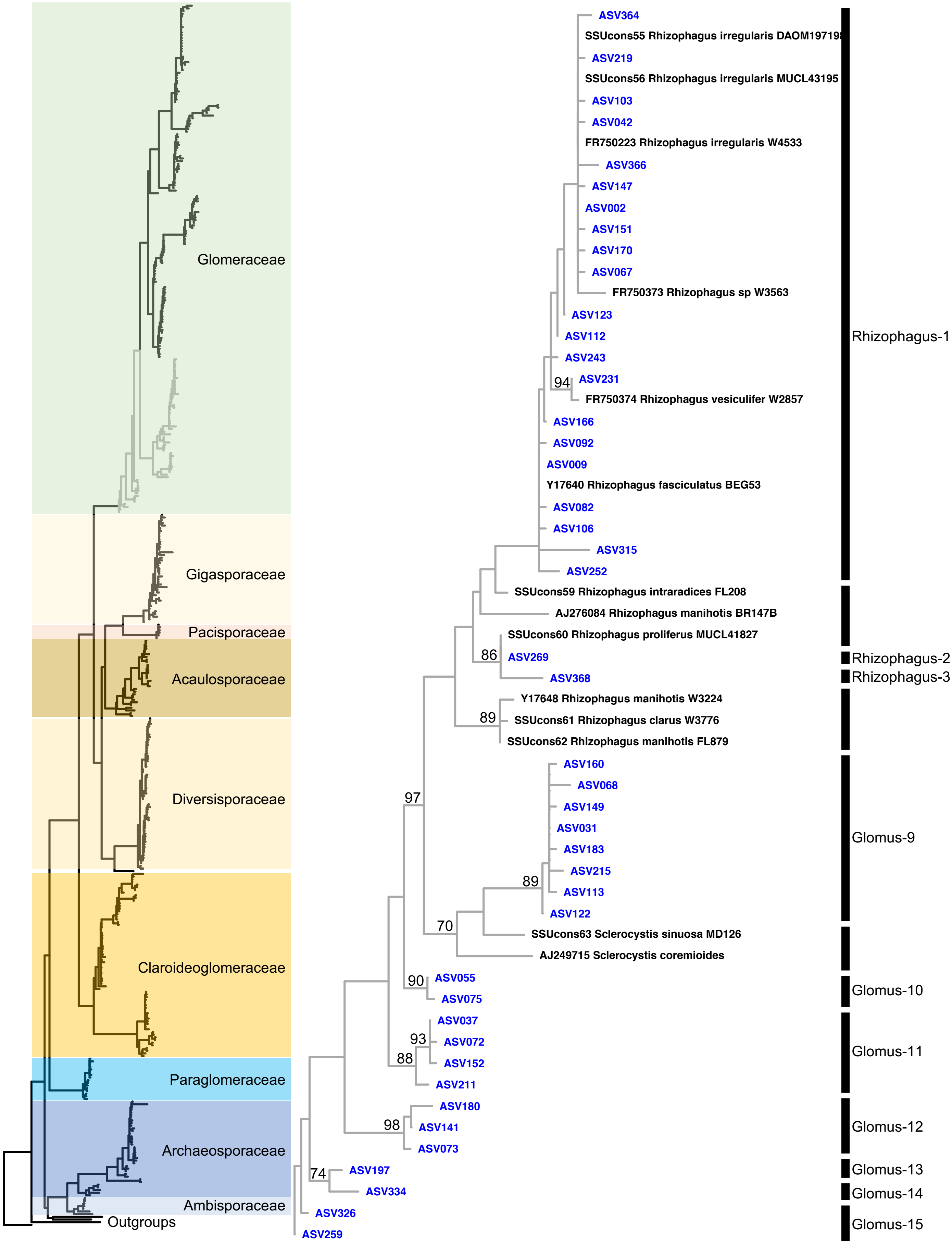
Glomeraceae
Gigasporaceae
Pacisporaceae
Acaulosporaceae
Diversisporaceae
Claroideoglomeraceae
Paraglomeraceae
Archaeosporaceae
Ambisporaceae
Outgroups
ASV364
SSUcons55 Rhizophagus irregularis DAOM19719
ASV219
SSUcons56 Rhizophagus irregularis MUCL43195
ASV103
ASV042
FR750223 Rhizophagus irregularis W4533
ASV366
ASV147
ASV002
ASV151
ASV170
ASV067
FR750373 Rhizophagus sp W3563
ASV123
ASV112
ASV243
94
ASV231
FR750374 Rhizophagus vesiculifer W2857
ASV166
ASV092
ASV009
Y17640 Rhizophagus fasciculatus BEG53
ASV082
ASV106
ASV315
ASV252
SSUcons59 Rhizophagus intraradices FL208
AJ276084 Rhizophagus manihotis BR147B
SSUcons60 Rhizophagus proliferus MUCL41827
86
ASV269
ASV368
Y17648 Rhizophagus manihotis W3224
89
SSUcons61 Rhizophagus clarus W3776
SSUcons62 Rhizophagus manihotis FL879
ASV160
ASV068
ASV149
ASV031
ASV183
ASV215
ASV113
ASV122
89
97
70
SSUcons63 Sclerocystis sinuosa MD126
AJ249715 Sclerocystis coremioides
90
ASV055
ASV075
ASV037
93
ASV072
88
ASV152
ASV211
ASV180
98
ASV141
ASV073
74
ASV197
ASV334
ASV326
ASV259
Rhizophagus-1
Rhizophagus-2
Rhizophagus-3
Glomus-9
Glomus-10
Glomus-11
Glomus-12
Glomus-13
Glomus-14
Glomus-15

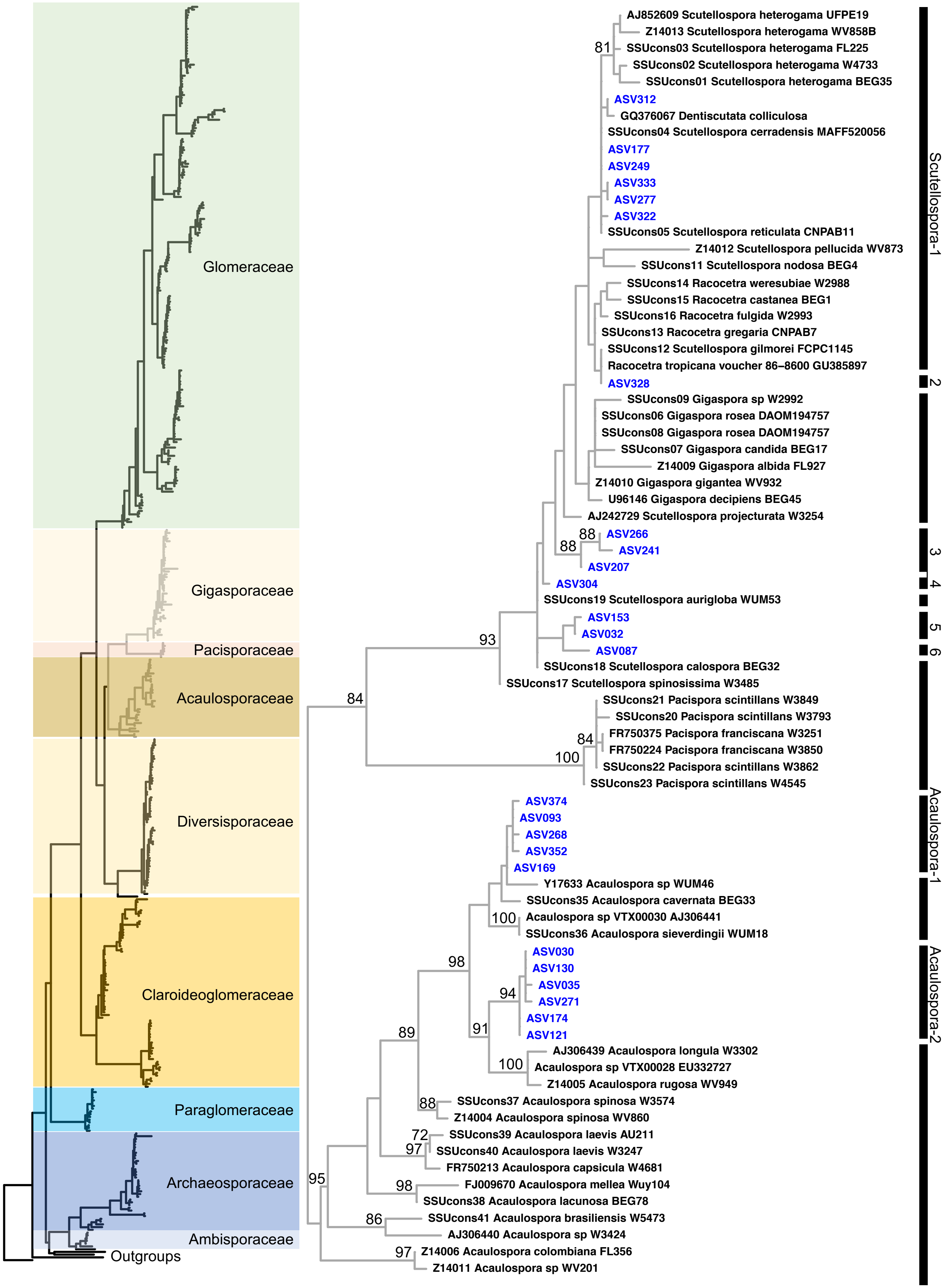
Glomeraceae
Gigasporaceae
Pacisporaceae
Acaulosporaceae
Diversisporaceae
Claroideoglomeraceae
Paraglomeraceae
Archaeosporaceae
Ambisporaceae
Outgroups
AJ852609 Scutellospora heterogama UFPE19
Z14013 Scutellospora heterogama WV858B
SSUcons03 Scutellospora heterogama FL225
SSUcons02 Scutellospora heterogama W4733
SSUcons01 Scutellospora heterogama BEG35
ASV312
GQ376067 Dentiscutata colliculosa
SSUcons04 Scutellospora cerradensis MAFF520056
ASV177
ASV249
ASV333
ASV277
ASV322
SSUcons05 Scutellospora reticulata CNPAB11
Z14012 Scutellospora pellucida WV873
SSUcons11 Scutellospora nodosa BEG4
SSUcons14 Racocetra weresubiae W2988
SSUcons15 Racocetra castanea BEG1
SSUcons16 Racocetra fulgida W2993
SSUcons13 Racocetra gregaria CNPAB7
SSUcons12 Scutellospora gilmorei FCPC1145
Racocetra tropicana voucher 86–8600 GU385897
ASV328
SSUcons09 Gigaspora sp W2992
SSUcons06 Gigaspora rosea DAOM194757
SSUcons08 Gigaspora rosea DAOM194757
SSUcons07 Gigaspora candida BEG17
Z14009 Gigaspora albida FL927
Z14010 Gigaspora gigantea WV932
U96146 Gigaspora decipiens BEG45
AJ242729 Scutellospora projecturata W3254
ASV266
ASV241
ASV207
ASV304
SSUcons19 Scutellospora aurigloba WUM53
ASV153
ASV032
ASV087
SSUcons18 Scutellospora calospora BEG32
SSUcons17 Scutellospora spinosissima W3485
SSUcons21 Pacispora scintillans W3849
SSUcons20 Pacispora scintillans W3793
FR750375 Pacispora franciscana W3251
FR750224 Pacispora franciscana W3850
SSUcons22 Pacispora scintillans W3862
SSUcons23 Pacispora scintillans W4545
ASV374
ASV093
ASV268
ASV352
ASV169
Y17633 Acaulospora sp WUM46
SSUcons35 Acaulospora cavernata BEG33
Acaulospora sp VTX00030 AJ306441
SSUcons36 Acaulospora sieverdingii WUM18
ASV030
ASV130
ASV035
ASV271
ASV174
ASV121
AJ306439 Acaulospora longula W3302
Acaulospora sp VTX00028 EU332727
Z14005 Acaulospora rugosa WV949
SSUcons37 Acaulospora spinosa W3574
Z14004 Acaulospora spinosa WV860
SSUcons39 Acaulospora laevis AU211
SSUcons40 Acaulospora laevis W3247
FR750213 Acaulospora capsicula W4681
FJ009670 Acaulospora mellea Wuy104
SSUcons38 Acaulospora lacunosa BEG78
SSUcons41 Acaulospora brasiliensis W5473
AJ306440 Acaulospora sp W3424
Z14006 Acaulospora colombiana FL356
Z14011 Acaulospora sp WV201
Scutellospora-1
2
3
4
5
6
Acaulospora-1
Acaulospora-2

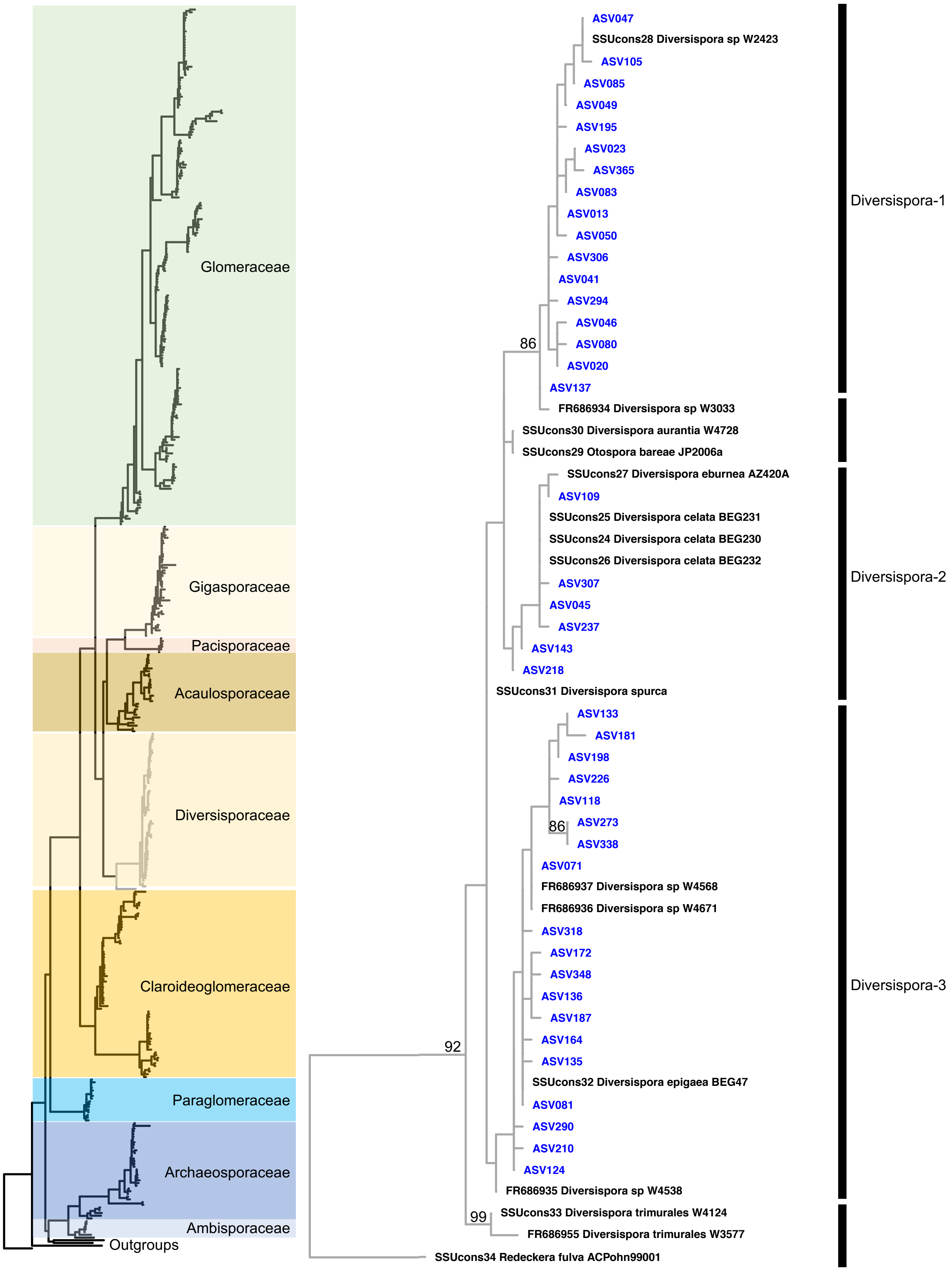

Glomeraceae
Gigasporaceae
Pacisporaceae
Acaulosporaceae
Diversisporaceae
Claroideoglomeraceae
Paraglomeraceae
Archaeosporaceae
Ambisporaceae
Outgroups
ASV047
SSUcons28 Diversispora sp W2423
ASV105
ASV085
ASV049
ASV195
ASV023
ASV365
ASV083
ASV013
ASV050
ASV306
ASV041
ASV294
ASV046
ASV080
ASV020
ASV137
86
FR686934 Diversispora sp W3033
SSUcons30 Diversispora aurantia W4728
SSUcons29 Otospora bareae JP2006a
SSUcons27 Diversispora eburnea AZ420A
ASV109
SSUcons25 Diversispora celata BEG231
SSUcons24 Diversispora celata BEG230
SSUcons26 Diversispora celata BEG232
ASV307
ASV045
ASV237
ASV143
ASV218
SSUcons31 Diversispora spurca
ASV133
ASV181
ASV198
ASV226
ASV118
86
ASV273
ASV338
ASV071
FR686937 Diversispora sp W4568
FR686936 Diversispora sp W4671
ASV318
ASV172
ASV348
ASV136
ASV187
ASV164
ASV135
SSUcons32 Diversispora epigaea BEG47
ASV081
ASV290
ASV210
ASV124
FR686935 Diversispora sp W4538
92
99
SSUcons33 Diversispora trimurales W4124
FR686955 Diversispora trimurales W3577
SSUcons34 Redeckera fulva ACPohn99001
Diversispora-1
Diversispora-2
Diversispora-3

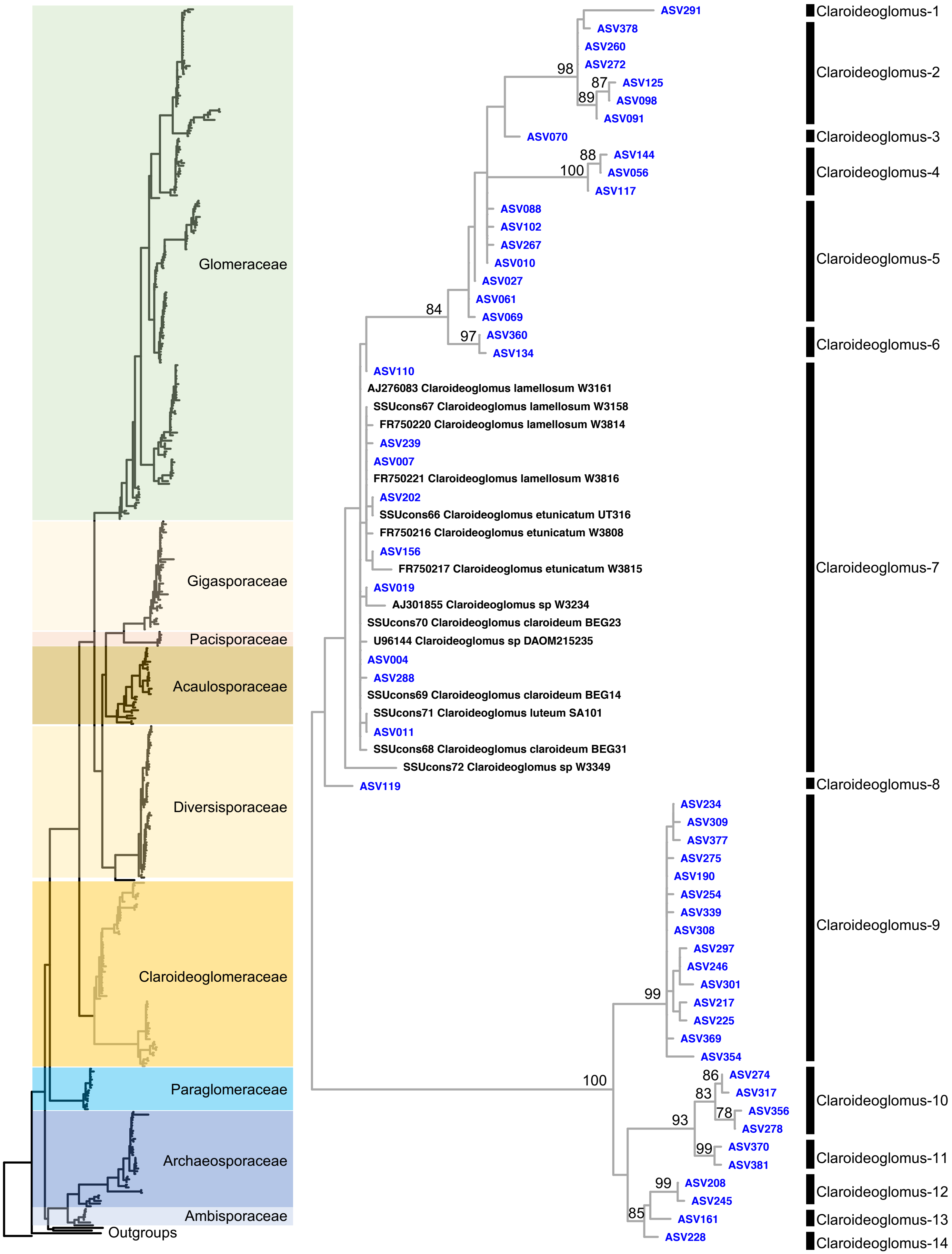
Glomeraceae
Gigasporaceae
Pacisporaceae
Acaulosporaceae
Diversisporaceae
Claroideoglomeraceae
Paraglomeraceae
Archaeosporaceae
Ambisporaceae
Outgroups
ASV291
ASV378
ASV260
ASV272
ASV125
ASV098
ASV091
ASV070
ASV144
ASV056
ASV117
ASV088
ASV102
ASV267
ASV010
ASV027
ASV061
ASV069
ASV360
ASV134
ASV110
AJ276083 Claroideoglomus lamellosum W3161
SSUcons67 Claroideoglomus lamellosum W3158
FR750220 Claroideoglomus lamellosum W3814
ASV239
ASV007
FR750221 Claroideoglomus lamellosum W3816
ASV202
SSUcons66 Claroideoglomus etunicatum UT316
FR750216 Claroideoglomus etunicatum W3808
ASV156
FR750217 Claroideoglomus etunicatum W3815
ASV019
AJ301855 Claroideoglomus sp W3234
SSUcons70 Claroideoglomus claroideum BEG23
U96144 Claroideoglomus sp DAOM215235
ASV004
ASV288
SSUcons69 Claroideoglomus claroideum BEG14
SSUcons71 Claroideoglomus luteum SA101
ASV011
SSUcons68 Claroideoglomus claroideum BEG31
SSUcons72 Claroideoglomus sp W3349
ASV119
ASV234
ASV309
ASV377
ASV275
ASV190
ASV254
ASV339
ASV308
ASV297
ASV246
ASV301
ASV217
ASV225
ASV369
ASV354
ASV274
ASV317
ASV356
ASV278
ASV370
ASV381
ASV208
ASV245
ASV161
ASV228
98
87
89
88
100
84
97
99
100
86
83
78
93
99
99
85
Claroideoglomus-1
Claroideoglomus-2
Claroideoglomus-3
Claroideoglomus-4
Claroideoglomus-5
Claroideoglomus-6
Claroideoglomus-7
Claroideoglomus-8
Claroideoglomus-9
Claroideoglomus-10
Claroideoglomus-11
Claroideoglomus-12
Claroideoglomus-13
Claroideoglomus-14

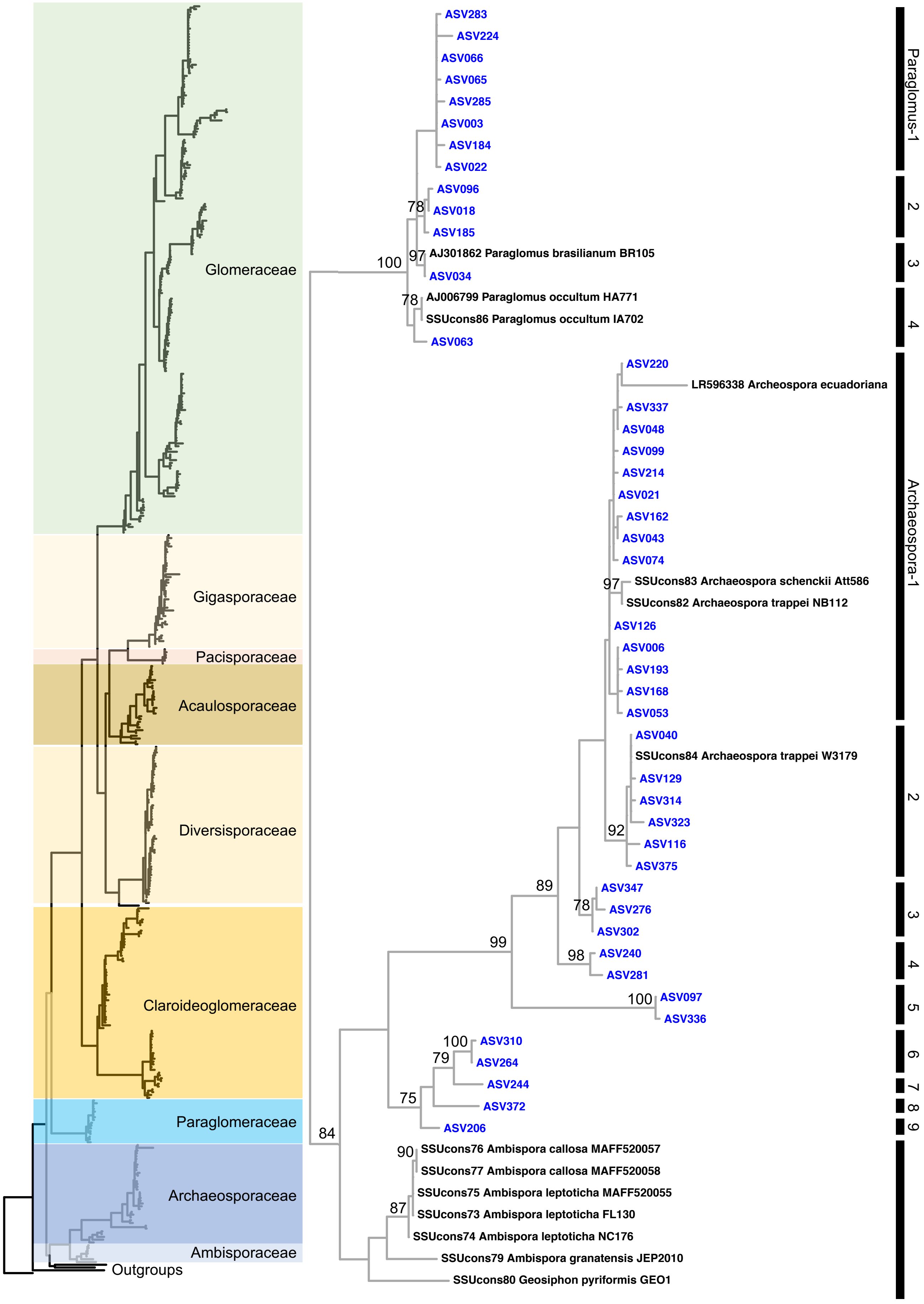

Glomeraceae
Gigasporaceae
Pacisporaceae
Acaulosporaceae
Diversisporaceae
Claroideoglomeraceae
Paraglomeraceae
Archaeosporaceae
Ambisporaceae
Outgroups
ASV283
ASV224
ASV066
ASV065
ASV285
ASV003
ASV184
ASV022
ASV096
ASV018
ASV185
AJ301862 Paraglomus brasilianum BR105
ASV034
AJ006799 Paraglomus occultum HA771
SSUcons86 Paraglomus occultum IA702
ASV063
ASV220
LR596338 Archeospora ecuadoriana
ASV337
ASV048
ASV099
ASV214
ASV021
ASV162
ASV043
ASV074
SSUcons83 Archaeospora schenckii Att586
SSUcons82 Archaeospora trappei NB112
ASV126
ASV006
ASV193
ASV168
ASV053
ASV040
SSUcons84 Archaeospora trappei W3179
ASV129
ASV314
ASV323
ASV116
ASV375
ASV347
ASV276
ASV302
ASV240
ASV281
ASV097
ASV336
ASV310
ASV264
ASV244
ASV372
ASV206
SSUcons76 Ambispora callosa MAFF520057
SSUcons77 Ambispora callosa MAFF520058
SSUcons75 Ambispora leptoticha MAFF520055
SSUcons73 Ambispora leptoticha FL130
SSUcons74 Ambispora leptoticha NC176
SSUcons79 Ambispora granatensis JEP2010
SSUcons80 Geosiphon pyriformis GEO1
Paraglomus-1
2
3
4
Archaeospora-1
2
3
4
5
6
7
8
9

Figure S3. RAxML phylogeny showing the taxonomic assignment of each amplicon sequence variant (ASV, shown in blue) to the family level (left) and genus level (right). Phylogenetic trees on the right side represent the clades coloured in grey in the phylogenetic tree on the left side. Only bootstrap values > 70 are shown. Within genus phylotypes have been determined using a clustering threshold of 99%.
