# Supplementary material for: Similar Arbuscular Mycorrhizal Fungal Communities in 31 Durum Wheat Cultivars (Triticum turgidum L. var. durum) Under Field Conditions in Eastern Canada
Source: Front Plant Sci. 2020 Aug 11;11:1206. doi: 10.3389/fpls.2020.01206 (PMC7431883; doi:10.3389/fpls.2020.01206)
Supplement: Supplementary file 2 [file Table_1.docx]

**Supplementary materials**

The main bioinformatic steps and its impact on the sequence dataset are summarized in Figure S2. Briefly, a total of 17,323,404 sequences (~ 450 bp) were obtained. The denoising procedure reduced the number of sequences to 5,441,927 for a total number of 1429 ASVs. This number was reduced to 910 following the elimination of rare ASVs. De novo clustering using a 100% threshold decreased the number of ASVs to 890 (see explanation in paragraph “Bioinformatic analyses”). Non-glomeromycotan sequences (191,016; 3.6%) were filtered, leading to an AMF dataset of 5,135,297 sequences distributed in 381 ASVs belonging to the phylum Glomeromycota. A visual inspection of the alignment of representative sequences combined with the analysis of a RAxML phylogenetic tree and BLAST search on NCBI allowed to detect and remove 49 chimeric sequences and 15 sequences featured by abnormal large deletions. Rarefaction curves (Figure S3A) showed that the ASV richness was saturated at a sequencing depth of 5000. Randomly subsampling each sample to 5000 sequences retained 1,680,000 (32.9%) sequences in 336 (90.3%) samples and 303 (95.6%) of the amplicon sequence variants (Figure S4).

The ADONIS permutation-based statistical test (as implemented in QIIME 2, 9999 permutations were run) was used to investigate whether the AMF communities recorded in soil samples extracted with the PowerSoil or Ultraclean kit were significantly different from one another. Results showed that only 6.6% of the sums of squares could be explained by the extraction kit while 7% of the sums of squares could be explained by the block (Table S4).

**Table S1 |** Physical and chemical properties of the soil from the experimental field. Analyses were performed in the spring of 2015. Mineral nitrogen was extracted with KCl (Maynard et al. 1993) and other nutrients were extracted with Mehlich-3 (Mehlich 1984).

| **Texture** | Loam |
| --- | --- |
| **pH** | 5.3 |
| **C total (%)** | 2.66 |
| **N total (%)** | 0.21 |
| **C/N** | 12.73 |
| **PO4-P (mg / kg)** | 34.52 |
| **NO3-N (mg / kg)** | 52.96 |
| **NH4-N (mg / kg)** | 43.32 |
| **P (mg / kg)** | 133.97 |
| **K (mg / kg)** | 205.11 |
| **Ca (mg / kg)** | 1632.19 |
| **Mg (mg / kg)** | 55.44 |
| **Al (mg / kg)** | 1346 |
| **Fe (mg / kg)** | 225.39 |
| **Cu (mg / kg)** | 1.74 |
| **Zn (mg / kg)** | 1.92 |
| **Mn (mg / kg)** | 15.92 |

Maynard, D.G., and Kalra, Y.P. 1993. Nitrate and extractable ammonium nitrogen. In M.R. Carter, Ed. Soil Sampling and Methods of Analysis. Lewis Publisher, Boca Raton, FL, pp. 25-38.

Mehlich, A. 1984. Mehlich-3 soil test extractant: a modification of Mehlich-2 extractant. *Commun. Soil. Sci. Plant Anal.* 15: 1409-1416.

**Table S2 |** Information about the five landraces (in bold) and 26 durum wheat cultivars seeded in the experimental field. The numbers in the study ID column allow to identify the distribution of cultivars in the field plots (see Figure S1).

| **Cultivar Name** | **Year of release / Registration date** | **Pedigree** | **Study ID** |
| --- | --- | --- | --- |
| **ARNAUTKA** | 1864 | LV-RUS; LV-ODESSA | 2 |
| **KUBANKA** | 1900 | LV | 11 |
| **MINDUM** | 1917 | (S)HEDGEROW | 16 |
| **GOLDEN BALL** | 1918 | (S)LV-SOUTH-AFRICA | 9 |
| **PELISSIER** | 1929 | (S)LV-DZA | 17 |
| RAMSEY | 1957 | CARLETON/(PAL)PI-94701 | 21 |
| LAKOTA | 1960 | SENTRY,USA//LD-379/LD-357 | 13 |
| STEWART 63 | 1963 | ST-464/8*STEWART,TR.DR; STEWART*8/ST-464 | 23 |
| HERCULES | 1969 | RL-3097/RL-3304//STEWART(TR.DR)/RL-3380 | 10 |
| QUILAFEN | 1970 | LD-357-E/2*TEHUACAN-60; E-5477//SENTRY/2*TEHUACAN-60 | 19 |
| WASCANA | 1971 | LAKOTA*2/PELISSIER | 25 |
| WAKOOMA | 1973 | LAKOTA*2/PELISSIER; LAKOTAPELISSIER; LAKOTA/2*PELISSIER | 24 |
| MACOUN | 1974 | RL-3607/DT-182 | 14 |
| MEDORA | 1982 | WARD/MACOUN | 15 |
| ARCOLA | 1983 | WASCANA/HERCULES | 1 |
| KYLE | 1984 | WAKOOMA/DT-322(6962-92-8-5)//(6965-494-1)WAKOOMA/DT-320 | 12 |
| SCEPTRE | 1985 | D-72110/COULTER | 20 |
| PLENTY | 1990 | VIC/WASCANA//DT-354 | 18 |
| AC MELITA | 1995 | MEDORA/LLOYD | 31 |
| AC AVONLEA | 1997 | SC-8267-AD-2A/DT-612 | 30 |
| AC MORSE | 1998 | RL-7196/D-84328 | 27 |
| AC PATHFINDER | 1998 | WESTBRED-881/DT-367; DT-367/WESTBRED-881 | 26 |
| AC NAPOLEON | 2001 | VIC/DT-384//DT-471 | 29 |
| AC NAVIGATOR | 2002 | KYLE/WESTBRED-881 | 28 |
| COMMANDER | 2004 | W-9260-BK-03/AC-NAVIGATOR//AC-PATHIFINDER | 5 |
| STRONGFIELD | 2004 | AC-AVONLEA/DT-665 | 22 |
| BRIGADE | 2008 | DT-513/DT-696 | 3 |
| CDC VERONA | 2008 | D-95253/D-95212 | 4 |
| EUROSTAR | 2008 | G-9575-B-AA-09-C/DT-498//DT-691 | 8 |
| ENTERPRISE | 2009 | DT-716/STRONGFIELD; 9488-C-CK-2/STRONGFIELD | 7 |
| TRANSCEND | 2010 | DT-707/DT-696 | 6 |

Source:

<https://www.inspection.gc.ca/active/netapp/regvar/regvar_resultse.aspx?lang=e&Reg=&Kind=Wheat&SubKind=Wheat%2C+Durum&Name=&PNTRadio=All&Rep=&Status=&startDate=&endDate=&btn_submit=Submit>

<https://wheat.pw.usda.gov/ggpages/gopher/cwc/CommWheatCult/cwc3.html>

<http://www.wheatpedigree.net>

**Table S3 |** List of soil samples extracted either with the UltraClean kit or with the PowerSoil kit.

| SampleID | Sample No. | Block | Microbiome | Genotype | Extraction Kit |
| --- | --- | --- | --- | --- | --- |
| E1 | 1 | 1 | Soil | AC_Melita | UltraClean |
| E2 | 2 | 1 | Soil | Eurostar | UltraClean |
| E3 | 3 | 1 | Soil | Lakota | UltraClean |
| E4 | 4 | 1 | Soil | Wascana | UltraClean |
| E5 | 5 | 1 | Soil | Stewart_63 | UltraClean |
| E6 | 6 | 1 | Soil | Macoun | UltraClean |
| E7 | 7 | 1 | Soil | Pelissier | UltraClean |
| E8 | 8 | 1 | Soil | AC_Napoleon | UltraClean |
| E9 | 9 | 1 | Soil | Arcola | UltraClean |
| E10 | 10 | 1 | Soil | Wakooma | UltraClean |
| E11 | 11 | 1 | Soil | Strongfield | UltraClean |
| E12 | 12 | 1 | Soil | Brigade | UltraClean |
| E13 | 13 | 1 | Soil | Ramsey | UltraClean |
| E14 | 14 | 1 | Soil | Golden Ball | UltraClean |
| E15 | 15 | 1 | Soil | Kyle | UltraClean |
| E16 | 16 | 1 | Soil | Mindum | UltraClean |
| E17 | 17 | 1 | Soil | Transcend_DT_801 | UltraClean |
| E18 | 18 | 1 | Soil | AC_Navigator | UltraClean |
| E19 | 19 | 1 | Soil | AC_Avonlea | UltraClean |
| E20 | 20 | 1 | Soil | AC_Pathfinder | UltraClean |
| E21 | 21 | 1 | Soil | Medora | UltraClean |
| E22 | 22 | 1 | Soil | Arnautka | UltraClean |
| E23 | 23 | 1 | Soil | Plenty | UltraClean |
| E24 | 24 | 1 | Soil | Enterprise | UltraClean |
| E25 | 25 | 1 | Soil | Sceptre | UltraClean |
| E26 | 26 | 1 | Soil | AC_Morse | UltraClean |
| E27 | 27 | 1 | Soil | Kubanka | UltraClean |
| E28 | 28 | 1 | Soil | Commander | UltraClean |
| E29 | 29 | 1 | Soil | Quilafen | UltraClean |
| E30 | 30 | 1 | Soil | CDC_Verona | UltraClean |
| E31 | 31 | 1 | Soil | Hercules | PowerSoil |
| E32 | 32 | 2 | Soil | Strongfield | PowerSoil |
| E33 | 33 | 2 | Soil | Kyle | UltraClean |
| E34 | 34 | 2 | Soil | Commander | PowerSoil |
| E35 | 35 | 2 | Soil | Mindum | PowerSoil |
| E36 | 36 | 2 | Soil | Brigade | PowerSoil |
| E37 | 37 | 2 | Soil | Arnautka | PowerSoil |
| E38 | 38 | 2 | Soil | AC_Napoleon | PowerSoil |
| E39 | 39 | 2 | Soil | Hercules | PowerSoil |
| E40 | 40 | 2 | Soil | AC_Pathfinder | UltraClean |
| E41 | 41 | 2 | Soil | Stewart_63 | UltraClean |
| E42 | 42 | 2 | Soil | Wakooma | UltraClean |
| E43 | 43 | 2 | Soil | Lakota | UltraClean |
| E44 | 44 | 2 | Soil | AC_Melita | UltraClean |
| E45 | 45 | 2 | Soil | AC_Avonlea | UltraClean |
| E46 | 46 | 2 | Soil | Golden Ball | UltraClean |
| E47 | 47 | 2 | Soil | CDC_Verona | UltraClean |
| E48 | 48 | 2 | Soil | Wascana | UltraClean |
| E49 | 49 | 2 | Soil | Eurostar | UltraClean |
| E50 | 50 | 2 | Soil | Sceptre | UltraClean |
| E51 | 51 | 2 | Soil | Plenty | UltraClean |
| E52 | 52 | 2 | Soil | Enterprise | UltraClean |
| E53 | 53 | 2 | Soil | Macoun | UltraClean |
| E54 | 54 | 2 | Soil | Kubanka | UltraClean |
| E55 | 55 | 2 | Soil | Pelissier | UltraClean |
| E56 | 56 | 2 | Soil | Ramsey | UltraClean |
| E57 | 57 | 2 | Soil | Arcola | UltraClean |
| E58 | 58 | 2 | Soil | Transcend_DT_801 | UltraClean |
| E59 | 59 | 2 | Soil | Quilafen | UltraClean |
| E60 | 60 | 2 | Soil | AC_Morse | UltraClean |
| E61 | 61 | 2 | Soil | Medora | UltraClean |
| E62 | 62 | 2 | Soil | AC_Navigator | UltraClean |
| E63 | 63 | 3 | Soil | Strongfield | UltraClean |
| E64 | 64 | 3 | Soil | Arnautka | UltraClean |
| E65 | 65 | 3 | Soil | AC_Pathfinder | UltraClean |
| E66 | 66 | 3 | Soil | Commander | UltraClean |
| E67 | 67 | 3 | Soil | Wakooma | UltraClean |
| E68 | 68 | 3 | Soil | CDC_Verona | UltraClean |
| E69 | 69 | 3 | Soil | Wascana | UltraClean |
| E70 | 70 | 3 | Soil | Transcend_DT_801 | UltraClean |
| E71 | 71 | 3 | Soil | Plenty | UltraClean |
| E72 | 72 | 3 | Soil | Kubanka | UltraClean |
| E73 | 73 | 3 | Soil | Enterprise | UltraClean |
| E74 | 74 | 3 | Soil | Pelissier | UltraClean |
| E75 | 75 | 3 | Soil | AC_Melita | UltraClean |
| E76 | 76 | 3 | Soil | Ramsey | UltraClean |
| E77 | 77 | 3 | Soil | Eurostar | UltraClean |
| E78 | 78 | 3 | Soil | Mindum | PowerSoil |
| E79 | 79 | 3 | Soil | Macoun | PowerSoil |
| E80 | 80 | 3 | Soil | Medora | PowerSoil |
| E81 | 81 | 3 | Soil | Stewart_63 | PowerSoil |
| E82 | 82 | 3 | Soil | Arcola | PowerSoil |
| E83 | 83 | 3 | Soil | Golden Ball | PowerSoil |
| E84 | 84 | 3 | Soil | Brigade | PowerSoil |
| E85 | 85 | 3 | Soil | AC_Navigator | PowerSoil |
| E86 | 86 | 3 | Soil | Sceptre | UltraClean |
| E87 | 87 | 3 | Soil | AC_Avonlea | PowerSoil |
| E88 | 88 | 3 | Soil | Quilafen | PowerSoil |
| E89 | 89 | 3 | Soil | Lakota | PowerSoil |
| E90 | 90 | 3 | Soil | Kyle | PowerSoil |
| E91 | 91 | 3 | Soil | AC_Morse | PowerSoil |
| E92 | 92 | 3 | Soil | AC_Napoleon | PowerSoil |
| E93 | 93 | 3 | Soil | Hercules | PowerSoil |
| E94 | 94 | 4 | Soil | Plenty | UltraClean |
| E95 | 95 | 4 | Soil | Ramsey | PowerSoil |
| E96 | 96 | 4 | Soil | AC_Morse | UltraClean |
| E97 | 97 | 4 | Soil | CDC_Verona | PowerSoil |
| E98 | 98 | 4 | Soil | Transcend_DT_801 | PowerSoil |
| E99 | 99 | 4 | Soil | Quilafen | UltraClean |
| E100 | 100 | 4 | Soil | AC_Navigator | PowerSoil |
| E101 | 101 | 4 | Soil | Kyle | UltraClean |
| E102 | 102 | 4 | Soil | Brigade | PowerSoil |
| E103 | 103 | 4 | Soil | Strongfield | PowerSoil |
| E104 | 104 | 4 | Soil | Commander | UltraClean |
| E105 | 105 | 4 | Soil | AC_Pathfinder | PowerSoil |
| E106 | 106 | 4 | Soil | Wakooma | PowerSoil |
| E107 | 107 | 4 | Soil | AC_Melita | PowerSoil |
| E108 | 108 | 4 | Soil | Eurostar | PowerSoil |
| E109 | 109 | 4 | Soil | Golden Ball | PowerSoil |
| E110 | 110 | 4 | Soil | Arcola | PowerSoil |
| E111 | 111 | 4 | Soil | Hercules | PowerSoil |
| E112 | 112 | 4 | Soil | Kubanka | PowerSoil |
| E113 | 113 | 4 | Soil | AC_Avonlea | PowerSoil |
| E114 | 114 | 4 | Soil | Mindum | PowerSoil |
| E115 | 115 | 4 | Soil | Pelissier | PowerSoil |
| E116 | 116 | 4 | Soil | Macoun | UltraClean |
| E117 | 117 | 4 | Soil | Lakota | PowerSoil |
| E118 | 118 | 4 | Soil | AC_Napoleon | PowerSoil |
| E119 | 119 | 4 | Soil | Medora | PowerSoil |
| E120 | 120 | 4 | Soil | Wascana | PowerSoil |
| E121 | 121 | 4 | Soil | Enterprise | PowerSoil |
| E122 | 122 | 4 | Soil | Stewart_63 | PowerSoil |
| E123 | 123 | 4 | Soil | Sceptre | PowerSoil |
| E124 | 124 | 4 | Soil | Arnautka | PowerSoil |

**Table S4 |** Results from Adonis test showing the proportion of variance (R2) explained by the factors “Extraction kit” and “Block” and their interaction.

|  | **Df** | **Sums of Sqs** | **Mean Sqs** | **F Model** | **R2** | **Pr(>F)** |
| --- | --- | --- | --- | --- | --- | --- |
| **Extraction Kit** | 1 | 0.789 | 0.789 | 8.036 | 0.0661 | 0.0001 |
| **Block** | 1 | 0.826 | 0.826 | 8.408 | 0.0692 | 0.0001 |
| **Extraction Kit:Block** | 1 | 0.205 | 0.205 | 2.088 | 0.0172 | 0.0293 |
| **Residuals** | 103 | 10.117 | 0.098 | — | 0.8475 | — |
| **Total** | 106 | 11.938 | — | — | 1 | — |

Table S5 | Thermodynamic features of the primer set designed for the nested PCR, calculated using the OligoAnalyzer tool from IDT website ([www.idtdna.com/calc/analyzer](http://www.idtdna.com/calc/analyzer)).

|  | **nu-SSU-0450-5'** | **nu-SSU-0899-3'** |
| --- | --- | --- |
| **Length** | 18 | 22 |
| **GC content (%)** | 50 | 31.8 |
| **Melting temperature (℃)** | 60.2 | 58.5 |
| **Hairpin (kcal mole^-1^)** | 0.58 | -0.59 |
| **Self-dimer-1** | Delta G: -5.36 kcal/mole \| Base Pairs:  4  5' CGCAAATTACCCAATCCC  : \|\|\|\| :  3' CCCTAACCCATTAAACGC | Delta G:  -5.36 kcal/mole \| Base Pairs:  6  5' ATAAATCCAAGAATTTCACCTC  \|\|\|\|  3' CTCCACTTTAAGAACCTAAATA |
| **Self-dimer-2** | Delta G:  -3.61 kcal/mole \| Base Pairs:  2  5' CGCAAATTACCCAATCCC  \|\|  3' CCCTAACCCATTAAACGC | Delta G:  -5.36 kcal/mole \| Base Pairs:  4  5' ATAAATCCAAGAATTTCACCTC  \|\|\|\| : : ::::  3' CTCCACTTTAAGAACCTAAATA |
| **Hetero-Dimer-1** | Delta G:  -7.31 kcal/mole \| Base Pairs:  5  5' CGCAAATTACCCAATCCC  \|\|\|\|\| ::  3' CTCCACTTTAAGAACCTAAATA |  |
| **Hetero-Dimer-2** | Delta G:  -3.42 kcal/mole \| Base Pairs:  3  5' CGCAAATTACCCAATCCC  : \|\|\|  3' CTCCACTTTAAGAACCTAAATA |  |

**Table S6** | Degenerate fusion primers used for the nested PCR. The Illumina adapters and primers are shown in black and red, respectively. These primers were used as a pool to amplify the V3-V4 fragment of the 18S rRNA genes during the second-round PCR. N, NN, and NNN are mixed sequence bases added to introduce sequence complexity.

| Primer name | Sequence (5’ – 3’) |
| --- | --- |
| Illu_Rd1_0N_nu-SSU-0450-5’ | TCGTCGGCAGCGTCAGATGTGTATAAGAGACAGCGCAAATTACCCAATCCC |
| Illu_Rd1_1N_nu-SSU-0450-5’ | TCGTCGGCAGCGTCAGATGTGTATAAGAGACAGNCGCAAATTACCCAATCCC |
| Illu_Rd1_2N_nu-SSU-0450-5’ | TCGTCGGCAGCGTCAGATGTGTATAAGAGACAGNNCGCAAATTACCCAATCCC |
| Illu_Rd1_3N_nu-SSU-0450-5’ | TCGTCGGCAGCGTCAGATGTGTATAAGAGACAGNNNCGCAAATTACCCAATCCC |
| Illu_Rd1_0N_nu-SSU-0899-3’ | GTCTCGTGGGCTCGGAGATGTGTATAAGAGACAGATAAATCCAAGAATTTCACCTC |
| Illu_Rd1_1N_nu-SSU-0899-3’ | GTCTCGTGGGCTCGGAGATGTGTATAAGAGACAGNATAAATCCAAGAATTTCACCTC |
| Illu_Rd1_2N_nu-SSU-0899-3’ | GTCTCGTGGGCTCGGAGATGTGTATAAGAGACAGNNATAAATCCAAGAATTTCACCTC |
| Illu_Rd1_3N_nu-SSU-0899-3’ | GTCTCGTGGGCTCGGAGATGTGTATAAGAGACAGNNNATAAATCCAAGAATTTCACCTC |

**Table S7 |** Taxonomic assignment of the 317 amplicon sequence variants (ASVs) based on the RAxML phylogenetic tree of Figure S5. ASVs are ranked from the most to the least abundant in terms of sequence number.

| **ASV** | **Phylum** | **Class** | **Order** | **Family** | **Genus** | **No. ASV per genus** |
| --- | --- | --- | --- | --- | --- | --- |
| ASV001 | Glomeromycota | Glomeromycetes | Glomerales | Glomeraceae | Funneliformis-1 | 14 |
| ASV002 | Glomeromycota | Glomeromycetes | Glomerales | Glomeraceae | Rhizophagus-1 | 21 |
| ASV003 | Glomeromycota | Paraglomeromycetes | Paraglomerales | Paraglomeraceae | Paraglomus-1 | 8 |
| ASV004 | Glomeromycota | Glomeromycetes | Glomerales | Claroideoglomeraceae | Claroideoglomus-7 | 9 |
| ASV005 | Glomeromycota | Glomeromycetes | Glomerales | Glomeraceae | Funneliformis-1 | 14 |
| ASV006 | Glomeromycota | Archaeosporomycetes | Archaeosporales | Archaeosporaceae | Archaeospora-1 | 14 |
| ASV007 | Glomeromycota | Glomeromycetes | Glomerales | Claroideoglomeraceae | Claroideoglomus-7 | 9 |
| ASV008 | Glomeromycota | Glomeromycetes | Glomerales | Glomeraceae | Funneliformis-2 | 5 |
| ASV009 | Glomeromycota | Glomeromycetes | Glomerales | Glomeraceae | Rhizophagus-1 | 21 |
| ASV010 | Glomeromycota | Glomeromycetes | Glomerales | Claroideoglomeraceae | Claroideoglomus-5 | 7 |
| ASV011 | Glomeromycota | Glomeromycetes | Glomerales | Claroideoglomeraceae | Claroideoglomus-7 | 9 |
| ASV012 | Glomeromycota | Glomeromycetes | Glomerales | Glomeraceae | Funneliformis-1 | 14 |
| ASV013 | Glomeromycota | Glomeromycetes | Diversisporales | Diversisporaceae | Diversispora-1 | 17 |
| ASV014 | Glomeromycota | Glomeromycetes | Glomerales | Glomeraceae | Dominikia-1 | 10 |
| ASV015 | Glomeromycota | Glomeromycetes | Glomerales | Glomeraceae | Funneliformis-2 | 5 |
| ASV016 | Glomeromycota | Glomeromycetes | Glomerales | Glomeraceae | Dominikia-2 | 2 |
| ASV017 | Glomeromycota | Glomeromycetes | Glomerales | Glomeraceae | Dominikia-3 | 6 |
| ASV018 | Glomeromycota | Paraglomeromycetes | Paraglomerales | Paraglomeraceae | Paraglomus-2 | 3 |
| ASV019 | Glomeromycota | Glomeromycetes | Glomerales | Claroideoglomeraceae | Claroideoglomus-7 | 9 |
| ASV020 | Glomeromycota | Glomeromycetes | Diversisporales | Diversisporaceae | Diversispora-1 | 17 |
| ASV021 | Glomeromycota | Archaeosporomycetes | Archaeosporales | Archaeosporaceae | Archaeospora-1 | 14 |
| ASV022 | Glomeromycota | Paraglomeromycetes | Paraglomerales | Paraglomeraceae | Paraglomus-1 | 8 |
| ASV023 | Glomeromycota | Glomeromycetes | Diversisporales | Diversisporaceae | Diversispora-1 | 17 |
| ASV024 | Glomeromycota | Glomeromycetes | Glomerales | Glomeraceae | Funneliformis-1 | 14 |
| ASV025 | Glomeromycota | Glomeromycetes | Glomerales | Glomeraceae | Dominikia-1 | 10 |
| ASV026 | Glomeromycota | Glomeromycetes | Glomerales | Glomeraceae | Dominikia-1 | 10 |
| ASV027 | Glomeromycota | Glomeromycetes | Glomerales | Claroideoglomeraceae | Claroideoglomus-5 | 7 |
| ASV028 | Glomeromycota | Glomeromycetes | Glomerales | Glomeraceae | Funneliformis-1 | 14 |
| ASV029 | Glomeromycota | Glomeromycetes | Glomerales | Glomeraceae | Dominikia-3 | 6 |
| ASV030 | Glomeromycota | Glomeromycetes | Diversisporales | Acaulosporaceae | Acaulospora-2 | 6 |
| ASV031 | Glomeromycota | Glomeromycetes | Glomerales | Glomeraceae | Glomus-9 | 8 |
| ASV032 | Glomeromycota | Glomeromycetes | Diversisporales | Gigasporaceae | Scutellospora-5 | 2 |
| ASV033 | Glomeromycota | Glomeromycetes | Glomerales | Glomeraceae | Dominikia-1 | 10 |
| ASV034 | Glomeromycota | Paraglomeromycetes | Paraglomerales | Paraglomeraceae | Paraglomus-3 | 1 |
| ASV035 | Glomeromycota | Glomeromycetes | Diversisporales | Acaulosporaceae | Acaulospora-2 | 6 |
| ASV036 | Glomeromycota | Glomeromycetes | Glomerales | Glomeraceae | Funneliformis-1 | 14 |
| ASV037 | Glomeromycota | Glomeromycetes | Glomerales | Glomeraceae | Glomus-11 | 4 |
| ASV039 | Glomeromycota | Glomeromycetes | Glomerales | Glomeraceae | Funneliformis-1 | 14 |
| ASV040 | Glomeromycota | Archaeosporomycetes | Archaeosporales | Archaeosporaceae | Archaeospora-2 | 6 |
| ASV041 | Glomeromycota | Glomeromycetes | Diversisporales | Diversisporaceae | Diversispora-1 | 17 |
| ASV042 | Glomeromycota | Glomeromycetes | Glomerales | Glomeraceae | Rhizophagus-1 | 21 |
| ASV043 | Glomeromycota | Archaeosporomycetes | Archaeosporales | Archaeosporaceae | Archaeospora-1 | 14 |
| ASV044 | Glomeromycota | Glomeromycetes | Glomerales | Glomeraceae | Funneliformis-2 | 5 |
| ASV045 | Glomeromycota | Glomeromycetes | Diversisporales | Diversisporaceae | Diversispora-2 | 6 |
| ASV046 | Glomeromycota | Glomeromycetes | Diversisporales | Diversisporaceae | Diversispora-1 | 17 |
| ASV047 | Glomeromycota | Glomeromycetes | Diversisporales | Diversisporaceae | Diversispora-1 | 17 |
| ASV048 | Glomeromycota | Archaeosporomycetes | Archaeosporales | Archaeosporaceae | Archaeospora-1 | 14 |
| ASV049 | Glomeromycota | Glomeromycetes | Diversisporales | Diversisporaceae | Diversispora-1 | 17 |
| ASV050 | Glomeromycota | Glomeromycetes | Diversisporales | Diversisporaceae | Diversispora-1 | 17 |
| ASV051 | Glomeromycota | Glomeromycetes | Glomerales | Glomeraceae | Dominikia-5 | 8 |
| ASV052 | Glomeromycota | Glomeromycetes | Glomerales | Glomeraceae | Funneliformis-1 | 14 |
| ASV053 | Glomeromycota | Archaeosporomycetes | Archaeosporales | Archaeosporaceae | Archaeospora-1 | 14 |
| ASV054 | Glomeromycota | Glomeromycetes | Glomerales | Glomeraceae | Dominikia-1 | 10 |
| ASV055 | Glomeromycota | Glomeromycetes | Glomerales | Glomeraceae | Glomus-10 | 2 |
| ASV056 | Glomeromycota | Glomeromycetes | Glomerales | Claroideoglomeraceae | Claroideoglomus-4 | 3 |
| ASV057 | Glomeromycota | Glomeromycetes | Glomerales | Glomeraceae | Glomus-7 | 4 |
| ASV058 | Glomeromycota | Glomeromycetes | Glomerales | Glomeraceae | Dominikia-6 | 27 |
| ASV059 | Glomeromycota | Glomeromycetes | Glomerales | Glomeraceae | Glomus-3 | 2 |
| ASV060 | Glomeromycota | Glomeromycetes | Glomerales | Glomeraceae | Dominikia-6 | 27 |
| ASV061 | Glomeromycota | Glomeromycetes | Glomerales | Claroideoglomeraceae | Claroideoglomus-5 | 7 |
| ASV062 | Glomeromycota | Glomeromycetes | Glomerales | Glomeraceae | Glomus-1 | 3 |
| ASV063 | Glomeromycota | Paraglomeromycetes | Paraglomerales | Paraglomeraceae | Paraglomus-4 | 1 |
| ASV064 | Glomeromycota | Glomeromycetes | Glomerales | Glomeraceae | Funneliformis-1 | 14 |
| ASV065 | Glomeromycota | Paraglomeromycetes | Paraglomerales | Paraglomeraceae | Paraglomus-1 | 8 |
| ASV066 | Glomeromycota | Paraglomeromycetes | Paraglomerales | Paraglomeraceae | Paraglomus-1 | 8 |
| ASV067 | Glomeromycota | Glomeromycetes | Glomerales | Glomeraceae | Rhizophagus-1 | 21 |
| ASV068 | Glomeromycota | Glomeromycetes | Glomerales | Glomeraceae | Glomus-9 | 8 |
| ASV069 | Glomeromycota | Glomeromycetes | Glomerales | Claroideoglomeraceae | Claroideoglomus-5 | 7 |
| ASV070 | Glomeromycota | Glomeromycetes | Glomerales | Claroideoglomeraceae | Claroideoglomus-3 | 1 |
| ASV071 | Glomeromycota | Glomeromycetes | Diversisporales | Diversisporaceae | Diversispora-3 | 19 |
| ASV072 | Glomeromycota | Glomeromycetes | Glomerales | Glomeraceae | Glomus-11 | 4 |
| ASV073 | Glomeromycota | Glomeromycetes | Glomerales | Glomeraceae | Glomus-12 | 3 |
| ASV074 | Glomeromycota | Archaeosporomycetes | Archaeosporales | Archaeosporaceae | Archaeospora-1 | 14 |
| ASV075 | Glomeromycota | Glomeromycetes | Glomerales | Glomeraceae | Glomus-10 | 2 |
| ASV077 | Glomeromycota | Glomeromycetes | Glomerales | Glomeraceae | Funneliformis-2 | 5 |
| ASV078 | Glomeromycota | Glomeromycetes | Glomerales | Glomeraceae | Funneliformis-2 | 5 |
| ASV079 | Glomeromycota | Glomeromycetes | Glomerales | Glomeraceae | Dominikia-1 | 10 |
| ASV080 | Glomeromycota | Glomeromycetes | Diversisporales | Diversisporaceae | Diversispora-1 | 17 |
| ASV081 | Glomeromycota | Glomeromycetes | Diversisporales | Diversisporaceae | Diversispora-3 | 19 |
| ASV082 | Glomeromycota | Glomeromycetes | Glomerales | Glomeraceae | Rhizophagus-1 | 21 |
| ASV083 | Glomeromycota | Glomeromycetes | Diversisporales | Diversisporaceae | Diversispora-1 | 17 |
| ASV084 | Glomeromycota | Glomeromycetes | Glomerales | Glomeraceae | Dominikia-5 | 8 |
| ASV085 | Glomeromycota | Glomeromycetes | Diversisporales | Diversisporaceae | Diversispora-1 | 17 |
| ASV087 | Glomeromycota | Glomeromycetes | Diversisporales | Gigasporaceae | Scutellospora-6 | 1 |
| ASV088 | Glomeromycota | Glomeromycetes | Glomerales | Claroideoglomeraceae | Claroideoglomus-5 | 7 |
| ASV089 | Glomeromycota | Glomeromycetes | Glomerales | Glomeraceae | Glomus-2 | 3 |
| ASV090 | Glomeromycota | Glomeromycetes | Glomerales | Glomeraceae | Dominikia-4 | 7 |
| ASV091 | Glomeromycota | Glomeromycetes | Glomerales | Claroideoglomeraceae | Claroideoglomus-2 | 6 |
| ASV092 | Glomeromycota | Glomeromycetes | Glomerales | Glomeraceae | Rhizophagus-1 | 21 |
| ASV093 | Glomeromycota | Glomeromycetes | Diversisporales | Acaulosporaceae | Acaulospora-1 | 5 |
| ASV094 | Glomeromycota | Glomeromycetes | Glomerales | Glomeraceae | Funneliformis-1 | 14 |
| ASV095 | Glomeromycota | Glomeromycetes | Glomerales | Glomeraceae | Dominikia-2 | 2 |
| ASV096 | Glomeromycota | Paraglomeromycetes | Paraglomerales | Paraglomeraceae | Paraglomus-2 | 3 |
| ASV097 | Glomeromycota | Archaeosporomycetes | Archaeosporales | Archaeosporaceae | Archaeospora-5 | 2 |
| ASV098 | Glomeromycota | Glomeromycetes | Glomerales | Claroideoglomeraceae | Claroideoglomus-2 | 6 |
| ASV099 | Glomeromycota | Archaeosporomycetes | Archaeosporales | Archaeosporaceae | Archaeospora-1 | 14 |
| ASV100 | Glomeromycota | Glomeromycetes | Glomerales | Glomeraceae | Dominikia-6 | 27 |
| ASV101 | Glomeromycota | Glomeromycetes | Glomerales | Glomeraceae | Glomus-1 | 3 |
| ASV102 | Glomeromycota | Glomeromycetes | Glomerales | Claroideoglomeraceae | Claroideoglomus-5 | 7 |
| ASV103 | Glomeromycota | Glomeromycetes | Glomerales | Glomeraceae | Rhizophagus-1 | 21 |
| ASV104 | Glomeromycota | Glomeromycetes | Glomerales | Glomeraceae | Glomus-7 | 4 |
| ASV105 | Glomeromycota | Glomeromycetes | Diversisporales | Diversisporaceae | Diversispora-1 | 17 |
| ASV106 | Glomeromycota | Glomeromycetes | Glomerales | Glomeraceae | Rhizophagus-1 | 21 |
| ASV107 | Glomeromycota | Glomeromycetes | Glomerales | Glomeraceae | Dominikia-3 | 6 |
| ASV109 | Glomeromycota | Glomeromycetes | Diversisporales | Diversisporaceae | Diversispora-2 | 6 |
| ASV110 | Glomeromycota | Glomeromycetes | Glomerales | Claroideoglomeraceae | Claroideoglomus-7 | 9 |
| ASV111 | Glomeromycota | Glomeromycetes | Glomerales | Glomeraceae | Dominikia-3 | 6 |
| ASV112 | Glomeromycota | Glomeromycetes | Glomerales | Glomeraceae | Rhizophagus-1 | 21 |
| ASV113 | Glomeromycota | Glomeromycetes | Glomerales | Glomeraceae | Glomus-9 | 8 |
| ASV114 | Glomeromycota | Glomeromycetes | Glomerales | Glomeraceae | Glomus-2 | 3 |
| ASV116 | Glomeromycota | Archaeosporomycetes | Archaeosporales | Archaeosporaceae | Archaeospora-2 | 6 |
| ASV117 | Glomeromycota | Glomeromycetes | Glomerales | Claroideoglomeraceae | Claroideoglomus-4 | 3 |
| ASV118 | Glomeromycota | Glomeromycetes | Diversisporales | Diversisporaceae | Diversispora-3 | 19 |
| ASV119 | Glomeromycota | Glomeromycetes | Glomerales | Claroideoglomeraceae | Claroideoglomus-8 | 1 |
| ASV121 | Glomeromycota | Glomeromycetes | Diversisporales | Acaulosporaceae | Acaulospora-2 | 6 |
| ASV122 | Glomeromycota | Glomeromycetes | Glomerales | Glomeraceae | Glomus-9 | 8 |
| ASV123 | Glomeromycota | Glomeromycetes | Glomerales | Glomeraceae | Rhizophagus-1 | 21 |
| ASV124 | Glomeromycota | Glomeromycetes | Diversisporales | Diversisporaceae | Diversispora-3 | 19 |
| ASV125 | Glomeromycota | Glomeromycetes | Glomerales | Claroideoglomeraceae | Claroideoglomus-2 | 6 |
| ASV126 | Glomeromycota | Archaeosporomycetes | Archaeosporales | Archaeosporaceae | Archaeospora-1 | 14 |
| ASV127 | Glomeromycota | Glomeromycetes | Glomerales | Glomeraceae | Septoglomus-1 | 2 |
| ASV128 | Glomeromycota | Glomeromycetes | Glomerales | Glomeraceae | Dominikia-6 | 27 |
| ASV129 | Glomeromycota | Archaeosporomycetes | Archaeosporales | Archaeosporaceae | Archaeospora-2 | 6 |
| ASV130 | Glomeromycota | Glomeromycetes | Diversisporales | Acaulosporaceae | Acaulospora-2 | 6 |
| ASV131 | Glomeromycota | Glomeromycetes | Glomerales | Glomeraceae | Dominikia-5 | 8 |
| ASV133 | Glomeromycota | Glomeromycetes | Diversisporales | Diversisporaceae | Diversispora-3 | 19 |
| ASV134 | Glomeromycota | Glomeromycetes | Glomerales | Claroideoglomeraceae | Claroideoglomus-6 | 2 |
| ASV135 | Glomeromycota | Glomeromycetes | Diversisporales | Diversisporaceae | Diversispora-3 | 19 |
| ASV136 | Glomeromycota | Glomeromycetes | Diversisporales | Diversisporaceae | Diversispora-3 | 19 |
| ASV137 | Glomeromycota | Glomeromycetes | Diversisporales | Diversisporaceae | Diversispora-1 | 17 |
| ASV138 | Glomeromycota | Glomeromycetes | Glomerales | Glomeraceae | Dominikia-6 | 27 |
| ASV139 | Glomeromycota | Glomeromycetes | Glomerales | Glomeraceae | Dominikia-6 | 27 |
| ASV140 | Glomeromycota | Glomeromycetes | Glomerales | Glomeraceae | Glomus-6 | 4 |
| ASV141 | Glomeromycota | Glomeromycetes | Glomerales | Glomeraceae | Glomus-12 | 3 |
| ASV142 | Glomeromycota | Glomeromycetes | Glomerales | Glomeraceae | Dominikia-5 | 8 |
| ASV143 | Glomeromycota | Glomeromycetes | Diversisporales | Diversisporaceae | Diversispora-2 | 6 |
| ASV144 | Glomeromycota | Glomeromycetes | Glomerales | Claroideoglomeraceae | Claroideoglomus-4 | 3 |
| ASV145 | Glomeromycota | Glomeromycetes | Glomerales | Glomeraceae | Glomus-6 | 4 |
| ASV146 | Glomeromycota | Glomeromycetes | Glomerales | Glomeraceae | Dominikia-6 | 27 |
| ASV147 | Glomeromycota | Glomeromycetes | Glomerales | Glomeraceae | Rhizophagus-1 | 21 |
| ASV148 | Glomeromycota | Glomeromycetes | Glomerales | Glomeraceae | Dominikia-6 | 27 |
| ASV149 | Glomeromycota | Glomeromycetes | Glomerales | Glomeraceae | Glomus-9 | 8 |
| ASV150 | Glomeromycota | Glomeromycetes | Glomerales | Glomeraceae | Glomus-4 | 1 |
| ASV151 | Glomeromycota | Glomeromycetes | Glomerales | Glomeraceae | Rhizophagus-1 | 21 |
| ASV152 | Glomeromycota | Glomeromycetes | Glomerales | Glomeraceae | Glomus-11 | 4 |
| ASV153 | Glomeromycota | Glomeromycetes | Diversisporales | Gigasporaceae | Scutellospora-5 | 2 |
| ASV155 | Glomeromycota | Glomeromycetes | Glomerales | Glomeraceae | Glomus-7 | 4 |
| ASV156 | Glomeromycota | Glomeromycetes | Glomerales | Claroideoglomeraceae | Claroideoglomus-7 | 9 |
| ASV157 | Glomeromycota | Glomeromycetes | Glomerales | Glomeraceae | Funneliformis-1 | 14 |
| ASV158 | Glomeromycota | Glomeromycetes | Glomerales | Glomeraceae | Dominikia-1 | 10 |
| ASV159 | Glomeromycota | Glomeromycetes | Glomerales | Glomeraceae | Dominikia-6 | 27 |
| ASV160 | Glomeromycota | Glomeromycetes | Glomerales | Glomeraceae | Glomus-9 | 8 |
| ASV161 | Glomeromycota | Glomeromycetes | Glomerales | Claroideoglomeraceae | Claroideoglomus-13 | 1 |
| ASV162 | Glomeromycota | Archaeosporomycetes | Archaeosporales | Archaeosporaceae | Archaeospora-1 | 14 |
| ASV164 | Glomeromycota | Glomeromycetes | Diversisporales | Diversisporaceae | Diversispora-3 | 19 |
| ASV166 | Glomeromycota | Glomeromycetes | Glomerales | Glomeraceae | Rhizophagus-1 | 21 |
| ASV167 | Glomeromycota | Glomeromycetes | Glomerales | Glomeraceae | Glomus-7 | 4 |
| ASV168 | Glomeromycota | Archaeosporomycetes | Archaeosporales | Archaeosporaceae | Archaeospora-1 | 14 |
| ASV169 | Glomeromycota | Glomeromycetes | Diversisporales | Acaulosporaceae | Acaulospora-1 | 5 |
| ASV170 | Glomeromycota | Glomeromycetes | Glomerales | Glomeraceae | Rhizophagus-1 | 21 |
| ASV172 | Glomeromycota | Glomeromycetes | Diversisporales | Diversisporaceae | Diversispora-3 | 19 |
| ASV173 | Glomeromycota | Glomeromycetes | Glomerales | Glomeraceae | Septoglomus-2 | 2 |
| ASV174 | Glomeromycota | Glomeromycetes | Diversisporales | Acaulosporaceae | Acaulospora-2 | 6 |
| ASV175 | Glomeromycota | Glomeromycetes | Glomerales | Glomeraceae | Dominikia-6 | 27 |
| ASV177 | Glomeromycota | Glomeromycetes | Diversisporales | Gigasporaceae | Scutellospora-1 | 6 |
| ASV179 | Glomeromycota | Glomeromycetes | Glomerales | Glomeraceae | Dominikia-4 | 7 |
| ASV180 | Glomeromycota | Glomeromycetes | Glomerales | Glomeraceae | Glomus-12 | 3 |
| ASV181 | Glomeromycota | Glomeromycetes | Diversisporales | Diversisporaceae | Diversispora-3 | 19 |
| ASV183 | Glomeromycota | Glomeromycetes | Glomerales | Glomeraceae | Glomus-9 | 8 |
| ASV184 | Glomeromycota | Paraglomeromycetes | Paraglomerales | Paraglomeraceae | Paraglomus-1 | 8 |
| ASV185 | Glomeromycota | Paraglomeromycetes | Paraglomerales | Paraglomeraceae | Paraglomus-2 | 3 |
| ASV186 | Glomeromycota | Glomeromycetes | Glomerales | Glomeraceae | Dominikia-6 | 27 |
| ASV187 | Glomeromycota | Glomeromycetes | Diversisporales | Diversisporaceae | Diversispora-3 | 19 |
| ASV188 | Glomeromycota | Glomeromycetes | Glomerales | Glomeraceae | Funneliformis-1 | 14 |
| ASV189 | Glomeromycota | Glomeromycetes | Glomerales | Glomeraceae | Dominikia-6 | 27 |
| ASV190 | Glomeromycota | Glomeromycetes | Glomerales | Claroideoglomeraceae | Claroideoglomus-9 | 15 |
| ASV191 | Glomeromycota | Glomeromycetes | Glomerales | Glomeraceae | Dominikia-6 | 27 |
| ASV192 | Glomeromycota | Glomeromycetes | Glomerales | Glomeraceae | Dominikia-4 | 7 |
| ASV193 | Glomeromycota | Archaeosporomycetes | Archaeosporales | Archaeosporaceae | Archaeospora-1 | 14 |
| ASV194 | Glomeromycota | Glomeromycetes | Glomerales | Glomeraceae | Dominikia-4 | 7 |
| ASV195 | Glomeromycota | Glomeromycetes | Diversisporales | Diversisporaceae | Diversispora-1 | 17 |
| ASV196 | Glomeromycota | Glomeromycetes | Glomerales | Glomeraceae | Dominikia-4 | 7 |
| ASV197 | Glomeromycota | Glomeromycetes | Glomerales | Glomeraceae | Glomus-13 | 1 |
| ASV198 | Glomeromycota | Glomeromycetes | Diversisporales | Diversisporaceae | Diversispora-3 | 19 |
| ASV199 | Glomeromycota | Glomeromycetes | Glomerales | Glomeraceae | Dominikia-6 | 27 |
| ASV201 | Glomeromycota | Glomeromycetes | Glomerales | Glomeraceae | Dominikia-5 | 8 |
| ASV202 | Glomeromycota | Glomeromycetes | Glomerales | Claroideoglomeraceae | Claroideoglomus-7 | 9 |
| ASV203 | Glomeromycota | Glomeromycetes | Glomerales | Glomeraceae | Dominikia-5 | 8 |
| ASV205 | Glomeromycota | Glomeromycetes | Glomerales | Glomeraceae | Dominikia-6 | 27 |
| ASV206 | Glomeromycota | Archaeosporomycetes | Archaeosporales | Archaeosporaceae | Archaeospora-9 | 1 |
| ASV207 | Glomeromycota | Glomeromycetes | Diversisporales | Gigasporaceae | Scutellospora-3 | 3 |
| ASV208 | Glomeromycota | Glomeromycetes | Glomerales | Claroideoglomeraceae | Claroideoglomus-12 | 2 |
| ASV209 | Glomeromycota | Glomeromycetes | Glomerales | Glomeraceae | Septoglomus-1 | 2 |
| ASV210 | Glomeromycota | Glomeromycetes | Diversisporales | Diversisporaceae | Diversispora-3 | 19 |
| ASV211 | Glomeromycota | Glomeromycetes | Glomerales | Glomeraceae | Glomus-11 | 4 |
| ASV212 | Glomeromycota | Glomeromycetes | Glomerales | Glomeraceae | Glomus-1 | 3 |
| ASV214 | Glomeromycota | Archaeosporomycetes | Archaeosporales | Archaeosporaceae | Archaeospora-1 | 14 |
| ASV215 | Glomeromycota | Glomeromycetes | Glomerales | Glomeraceae | Glomus-9 | 8 |
| ASV216 | Glomeromycota | Glomeromycetes | Glomerales | Glomeraceae | Septoglomus-3 | 1 |
| ASV217 | Glomeromycota | Glomeromycetes | Glomerales | Claroideoglomeraceae | Claroideoglomus-9 | 15 |
| ASV218 | Glomeromycota | Glomeromycetes | Diversisporales | Diversisporaceae | Diversispora-2 | 6 |
| ASV219 | Glomeromycota | Glomeromycetes | Glomerales | Glomeraceae | Rhizophagus-1 | 21 |
| ASV220 | Glomeromycota | Archaeosporomycetes | Archaeosporales | Archaeosporaceae | Archaeospora-1 | 14 |
| ASV221 | Glomeromycota | Glomeromycetes | Glomerales | Glomeraceae | Dominikia-6 | 27 |
| ASV224 | Glomeromycota | Paraglomeromycetes | Paraglomerales | Paraglomeraceae | Paraglomus-1 | 8 |
| ASV225 | Glomeromycota | Glomeromycetes | Glomerales | Claroideoglomeraceae | Claroideoglomus-9 | 15 |
| ASV226 | Glomeromycota | Glomeromycetes | Diversisporales | Diversisporaceae | Diversispora-3 | 19 |
| ASV227 | Glomeromycota | Glomeromycetes | Glomerales | Glomeraceae | Dominikia-6 | 27 |
| ASV228 | Glomeromycota | Glomeromycetes | Glomerales | Claroideoglomeraceae | Claroideoglomus-14 | 1 |
| ASV231 | Glomeromycota | Glomeromycetes | Glomerales | Glomeraceae | Rhizophagus-1 | 21 |
| ASV232 | Glomeromycota | Glomeromycetes | Glomerales | Glomeraceae | Dominikia-6 | 27 |
| ASV233 | Glomeromycota | Glomeromycetes | Glomerales | Glomeraceae | Dominikia-6 | 27 |
| ASV234 | Glomeromycota | Glomeromycetes | Glomerales | Claroideoglomeraceae | Claroideoglomus-9 | 15 |
| ASV235 | Glomeromycota | Glomeromycetes | Glomerales | Glomeraceae | Dominikia-4 | 7 |
| ASV236 | Glomeromycota | Glomeromycetes | Glomerales | Glomeraceae | Dominikia-6 | 27 |
| ASV237 | Glomeromycota | Glomeromycetes | Diversisporales | Diversisporaceae | Diversispora-2 | 6 |
| ASV239 | Glomeromycota | Glomeromycetes | Glomerales | Claroideoglomeraceae | Claroideoglomus-7 | 9 |
| ASV240 | Glomeromycota | Archaeosporomycetes | Archaeosporales | Archaeosporaceae | Archaeospora-4 | 2 |
| ASV241 | Glomeromycota | Glomeromycetes | Diversisporales | Gigasporaceae | Scutellospora-3 | 3 |
| ASV243 | Glomeromycota | Glomeromycetes | Glomerales | Glomeraceae | Rhizophagus-1 | 21 |
| ASV244 | Glomeromycota | Archaeosporomycetes | Archaeosporales | Archaeosporaceae | Archaeospora-7 | 1 |
| ASV245 | Glomeromycota | Glomeromycetes | Glomerales | Claroideoglomeraceae | Claroideoglomus-12 | 2 |
| ASV246 | Glomeromycota | Glomeromycetes | Glomerales | Claroideoglomeraceae | Claroideoglomus-9 | 15 |
| ASV247 | Glomeromycota | Glomeromycetes | Glomerales | Glomeraceae | Glomus-3 | 2 |
| ASV249 | Glomeromycota | Glomeromycetes | Diversisporales | Gigasporaceae | Scutellospora-1 | 6 |
| ASV252 | Glomeromycota | Glomeromycetes | Glomerales | Glomeraceae | Rhizophagus-1 | 21 |
| ASV254 | Glomeromycota | Glomeromycetes | Glomerales | Claroideoglomeraceae | Claroideoglomus-9 | 15 |
| ASV255 | Glomeromycota | Glomeromycetes | Glomerales | Glomeraceae | Septoglomus-4 | 1 |
| ASV256 | Glomeromycota | Glomeromycetes | Glomerales | Glomeraceae | Dominikia-3 | 6 |
| ASV257 | Glomeromycota | Glomeromycetes | Glomerales | Glomeraceae | Dominikia-6 | 27 |
| ASV259 | Glomeromycota | Glomeromycetes | Glomerales | Glomeraceae | Glomus-15 | 2 |
| ASV260 | Glomeromycota | Glomeromycetes | Glomerales | Claroideoglomeraceae | Claroideoglomus-2 | 6 |
| ASV261 | Glomeromycota | Glomeromycetes | Glomerales | Glomeraceae | Dominikia-6 | 27 |
| ASV262 | Glomeromycota | Glomeromycetes | Glomerales | Glomeraceae | Dominikia-6 | 27 |
| ASV263 | Glomeromycota | Glomeromycetes | Glomerales | Glomeraceae | Glomus-5 | 1 |
| ASV264 | Glomeromycota | Archaeosporomycetes | Archaeosporales | Archaeosporaceae | Archaeospora-6 | 2 |
| ASV266 | Glomeromycota | Glomeromycetes | Diversisporales | Gigasporaceae | Scutellospora-3 | 3 |
| ASV267 | Glomeromycota | Glomeromycetes | Glomerales | Claroideoglomeraceae | Claroideoglomus-5 | 7 |
| ASV268 | Glomeromycota | Glomeromycetes | Diversisporales | Acaulosporaceae | Acaulospora-1 | 5 |
| ASV269 | Glomeromycota | Glomeromycetes | Glomerales | Glomeraceae | Rhizophagus-2 | 1 |
| ASV270 | Glomeromycota | Glomeromycetes | Glomerales | Glomeraceae | Glomus-6 | 4 |
| ASV271 | Glomeromycota | Glomeromycetes | Diversisporales | Acaulosporaceae | Acaulospora-2 | 6 |
| ASV272 | Glomeromycota | Glomeromycetes | Glomerales | Claroideoglomeraceae | Claroideoglomus-2 | 6 |
| ASV273 | Glomeromycota | Glomeromycetes | Diversisporales | Diversisporaceae | Diversispora-3 | 19 |
| ASV274 | Glomeromycota | Glomeromycetes | Glomerales | Claroideoglomeraceae | Claroideoglomus-10 | 4 |
| ASV275 | Glomeromycota | Glomeromycetes | Glomerales | Claroideoglomeraceae | Claroideoglomus-9 | 15 |
| ASV276 | Glomeromycota | Archaeosporomycetes | Archaeosporales | Archaeosporaceae | Archaeospora-3 | 3 |
| ASV277 | Glomeromycota | Glomeromycetes | Diversisporales | Gigasporaceae | Scutellospora-1 | 6 |
| ASV278 | Glomeromycota | Glomeromycetes | Glomerales | Claroideoglomeraceae | Claroideoglomus-10 | 4 |
| ASV280 | Glomeromycota | Glomeromycetes | Glomerales | Glomeraceae | Dominikia-1 | 10 |
| ASV281 | Glomeromycota | Archaeosporomycetes | Archaeosporales | Archaeosporaceae | Archaeospora-4 | 2 |
| ASV283 | Glomeromycota | Paraglomeromycetes | Paraglomerales | Paraglomeraceae | Paraglomus-1 | 8 |
| ASV285 | Glomeromycota | Paraglomeromycetes | Paraglomerales | Paraglomeraceae | Paraglomus-1 | 8 |
| ASV288 | Glomeromycota | Glomeromycetes | Glomerales | Claroideoglomeraceae | Claroideoglomus-7 | 9 |
| ASV290 | Glomeromycota | Glomeromycetes | Diversisporales | Diversisporaceae | Diversispora-3 | 19 |
| ASV291 | Glomeromycota | Glomeromycetes | Glomerales | Claroideoglomeraceae | Claroideoglomus-1 | 1 |
| ASV292 | Glomeromycota | Glomeromycetes | Glomerales | Glomeraceae | Dominikia-6 | 27 |
| ASV294 | Glomeromycota | Glomeromycetes | Diversisporales | Diversisporaceae | Diversispora-1 | 17 |
| ASV296 | Glomeromycota | Glomeromycetes | Glomerales | Glomeraceae | Dominikia-6 | 27 |
| ASV297 | Glomeromycota | Glomeromycetes | Glomerales | Claroideoglomeraceae | Claroideoglomus-9 | 15 |
| ASV299 | Glomeromycota | Glomeromycetes | Glomerales | Glomeraceae | Dominikia-4 | 7 |
| ASV300 | Glomeromycota | Glomeromycetes | Glomerales | Glomeraceae | Dominikia-6 | 27 |
| ASV301 | Glomeromycota | Glomeromycetes | Glomerales | Claroideoglomeraceae | Claroideoglomus-9 | 15 |
| ASV302 | Glomeromycota | Archaeosporomycetes | Archaeosporales | Archaeosporaceae | Archaeospora-3 | 3 |
| ASV304 | Glomeromycota | Glomeromycetes | Diversisporales | Gigasporaceae | Scutellospora-4 | 1 |
| ASV306 | Glomeromycota | Glomeromycetes | Diversisporales | Diversisporaceae | Diversispora-1 | 17 |
| ASV307 | Glomeromycota | Glomeromycetes | Diversisporales | Diversisporaceae | Diversispora-2 | 6 |
| ASV308 | Glomeromycota | Glomeromycetes | Glomerales | Claroideoglomeraceae | Claroideoglomus-9 | 15 |
| ASV309 | Glomeromycota | Glomeromycetes | Glomerales | Claroideoglomeraceae | Claroideoglomus-9 | 15 |
| ASV310 | Glomeromycota | Archaeosporomycetes | Archaeosporales | Archaeosporaceae | Archaeospora-6 | 2 |
| ASV311 | Glomeromycota | Glomeromycetes | Glomerales | Glomeraceae | Dominikia-3 | 6 |
| ASV312 | Glomeromycota | Glomeromycetes | Diversisporales | Gigasporaceae | Scutellospora-1 | 6 |
| ASV314 | Glomeromycota | Archaeosporomycetes | Archaeosporales | Archaeosporaceae | Archaeospora-2 | 6 |
| ASV315 | Glomeromycota | Glomeromycetes | Glomerales | Glomeraceae | Rhizophagus-1 | 21 |
| ASV317 | Glomeromycota | Glomeromycetes | Glomerales | Claroideoglomeraceae | Claroideoglomus-10 | 4 |
| ASV318 | Glomeromycota | Glomeromycetes | Diversisporales | Diversisporaceae | Diversispora-3 | 19 |
| ASV319 | Glomeromycota | Glomeromycetes | Glomerales | Glomeraceae | Septoglomus-5 | 2 |
| ASV322 | Glomeromycota | Glomeromycetes | Diversisporales | Gigasporaceae | Scutellospora-1 | 6 |
| ASV323 | Glomeromycota | Archaeosporomycetes | Archaeosporales | Archaeosporaceae | Archaeospora-2 | 6 |
| ASV324 | Glomeromycota | Glomeromycetes | Glomerales | Glomeraceae | Glomus-2 | 3 |
| ASV325 | Glomeromycota | Glomeromycetes | Glomerales | Glomeraceae | Septoglomus-2 | 2 |
| ASV326 | Glomeromycota | Glomeromycetes | Glomerales | Glomeraceae | Glomus-15 | 2 |
| ASV328 | Glomeromycota | Glomeromycetes | Diversisporales | Gigasporaceae | Scutellospora-2 | 1 |
| ASV330 | Glomeromycota | Glomeromycetes | Glomerales | Glomeraceae | Dominikia-6 | 27 |
| ASV333 | Glomeromycota | Glomeromycetes | Diversisporales | Gigasporaceae | Scutellospora-1 | 6 |
| ASV334 | Glomeromycota | Glomeromycetes | Glomerales | Glomeraceae | Glomus-14 | 1 |
| ASV335 | Glomeromycota | Glomeromycetes | Glomerales | Glomeraceae | Dominikia-5 | 8 |
| ASV336 | Glomeromycota | Archaeosporomycetes | Archaeosporales | Archaeosporaceae | Archaeospora-5 | 2 |
| ASV337 | Glomeromycota | Archaeosporomycetes | Archaeosporales | Archaeosporaceae | Archaeospora-1 | 14 |
| ASV338 | Glomeromycota | Glomeromycetes | Diversisporales | Diversisporaceae | Diversispora-3 | 19 |
| ASV339 | Glomeromycota | Glomeromycetes | Glomerales | Claroideoglomeraceae | Claroideoglomus-9 | 15 |
| ASV343 | Glomeromycota | Glomeromycetes | Glomerales | Glomeraceae | Glomus-6 | 4 |
| ASV344 | Glomeromycota | Glomeromycetes | Glomerales | Glomeraceae | Septoglomus-5 | 2 |
| ASV347 | Glomeromycota | Archaeosporomycetes | Archaeosporales | Archaeosporaceae | Archaeospora-3 | 3 |
| ASV348 | Glomeromycota | Glomeromycetes | Diversisporales | Diversisporaceae | Diversispora-3 | 19 |
| ASV349 | Glomeromycota | Glomeromycetes | Glomerales | Glomeraceae | Dominikia-1 | 10 |
| ASV350 | Glomeromycota | Glomeromycetes | Glomerales | Glomeraceae | Glomus-8 | 1 |
| ASV352 | Glomeromycota | Glomeromycetes | Diversisporales | Acaulosporaceae | Acaulospora-1 | 5 |
| ASV353 | Glomeromycota | Glomeromycetes | Glomerales | Glomeraceae | Dominikia-1 | 10 |
| ASV354 | Glomeromycota | Glomeromycetes | Glomerales | Claroideoglomeraceae | Claroideoglomus-9 | 15 |
| ASV355 | Glomeromycota | Glomeromycetes | Glomerales | Glomeraceae | Funneliformis-1 | 14 |
| ASV356 | Glomeromycota | Glomeromycetes | Glomerales | Claroideoglomeraceae | Claroideoglomus-10 | 4 |
| ASV360 | Glomeromycota | Glomeromycetes | Glomerales | Claroideoglomeraceae | Claroideoglomus-6 | 2 |
| ASV364 | Glomeromycota | Glomeromycetes | Glomerales | Glomeraceae | Rhizophagus-1 | 21 |
| ASV365 | Glomeromycota | Glomeromycetes | Diversisporales | Diversisporaceae | Diversispora-1 | 17 |
| ASV366 | Glomeromycota | Glomeromycetes | Glomerales | Glomeraceae | Rhizophagus-1 | 21 |
| ASV367 | Glomeromycota | Glomeromycetes | Glomerales | Glomeraceae | Funneliformis-1 | 14 |
| ASV368 | Glomeromycota | Glomeromycetes | Glomerales | Glomeraceae | Rhizophagus-3 | 1 |
| ASV369 | Glomeromycota | Glomeromycetes | Glomerales | Claroideoglomeraceae | Claroideoglomus-9 | 15 |
| ASV370 | Glomeromycota | Glomeromycetes | Glomerales | Claroideoglomeraceae | Claroideoglomus-11 | 2 |
| ASV372 | Glomeromycota | Archaeosporomycetes | Archaeosporales | Archaeosporaceae | Archaeospora-8 | 1 |
| ASV374 | Glomeromycota | Glomeromycetes | Diversisporales | Acaulosporaceae | Acaulospora-1 | 5 |
| ASV375 | Glomeromycota | Archaeosporomycetes | Archaeosporales | Archaeosporaceae | Archaeospora-2 | 6 |
| ASV377 | Glomeromycota | Glomeromycetes | Glomerales | Claroideoglomeraceae | Claroideoglomus-9 | 15 |
| ASV378 | Glomeromycota | Glomeromycetes | Glomerales | Claroideoglomeraceae | Claroideoglomus-2 | 6 |
| ASV379 | Glomeromycota | Glomeromycetes | Glomerales | Glomeraceae | Dominikia-5 | 8 |
| ASV381 | Glomeromycota | Glomeromycetes | Glomerales | Claroideoglomeraceae | Claroideoglomus-11 | 2 |

**Table S8 |** Cophenetic correlation matrix showing the level of similarity between the dendrograms from the Bray-Curtis dissimilarity analysis of the core ASVs recorded in soil, rhizosphere and root compartments (see dendrograms on top of heatmaps in Figure 4).

|  | Soil | Rhizosphere |
| --- | --- | --- |
| Rhizosphere | 0.03 | ⎯ |
| Root | -0.09 | 0.03 |

**
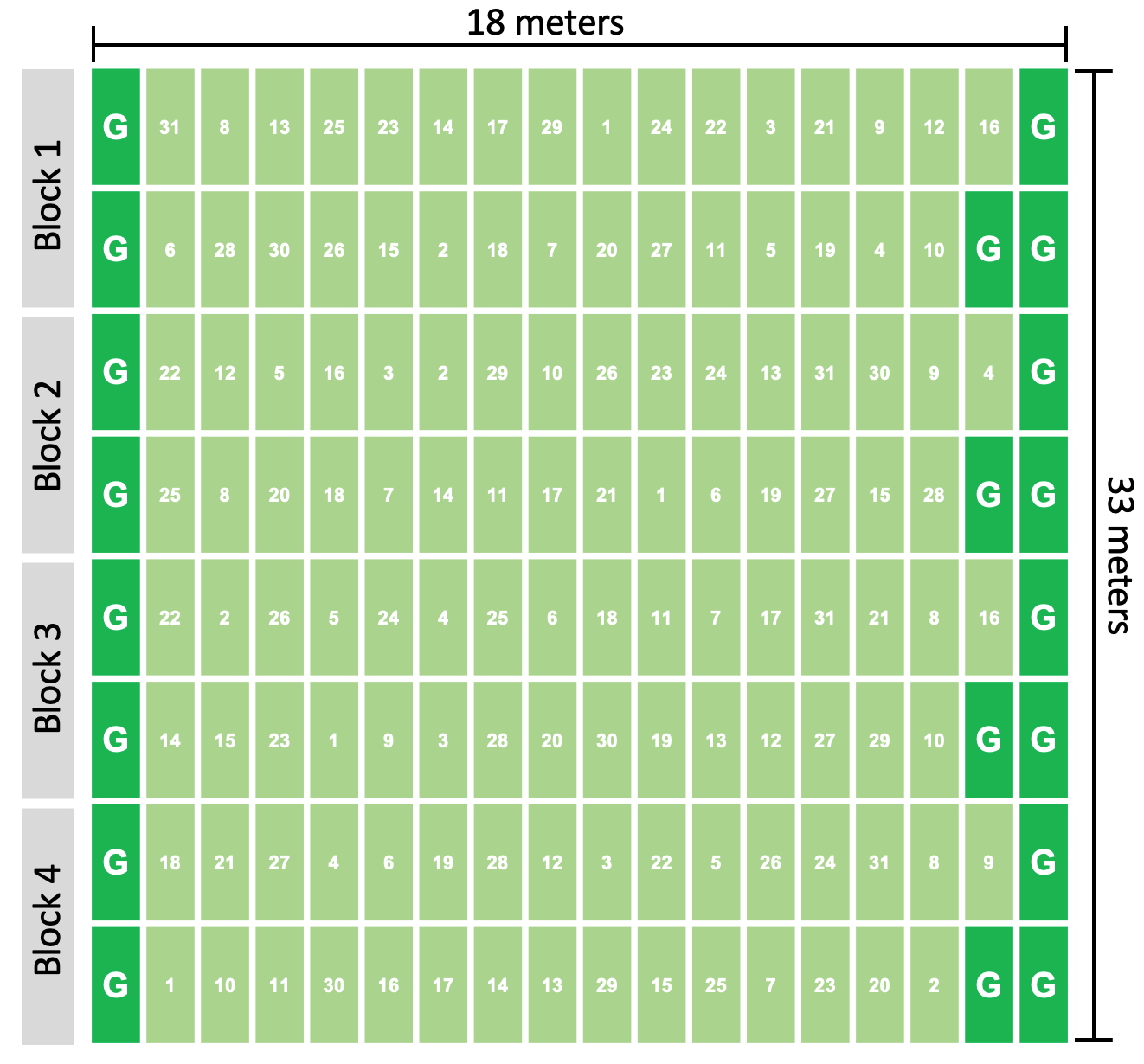
Figure S1.** Schematic representation of the experimental set-up of the field trial. A randomized complete block design of four blocks of 36 plots was conducted in summer 2016. The numbers inside each plot represent the cultivar ID numbers (1-31, correspondence to cultivar is provided in Table S2). Each plot (light green) had four rows and were each seeded with 50 grains. Plot size was 1 m x 1.5 m. Due to the large number of treatments and the dimension of the field where the experiment was conducted, each block was layered in two rows of plots. A guard plot (G, dark green) was seeded with cultivar AAC Cabri at both ends of each row.


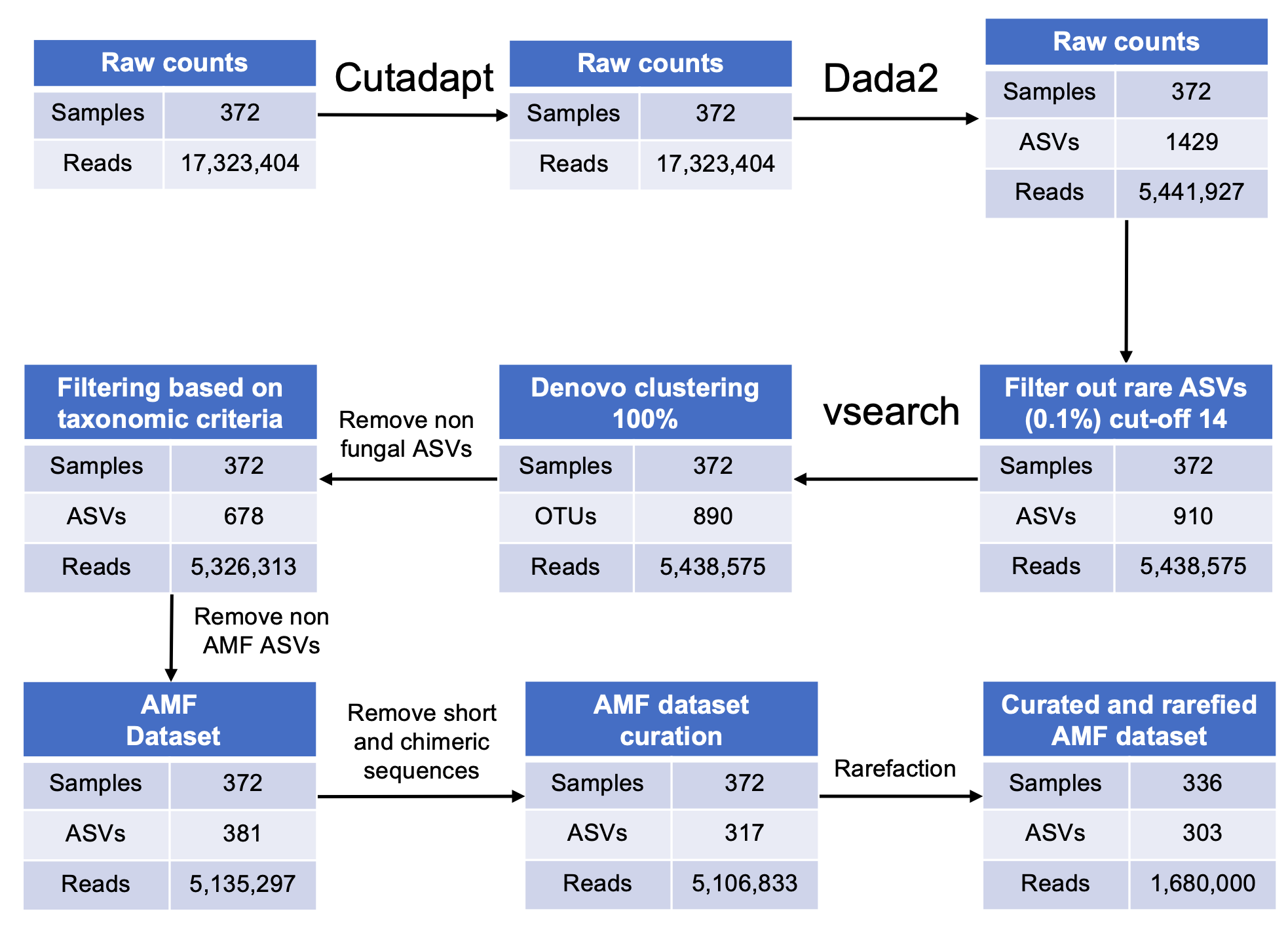
**Figure S2.** Bioinformatic workflow showing the impact of each step on the number of samples, reads and ASVs.


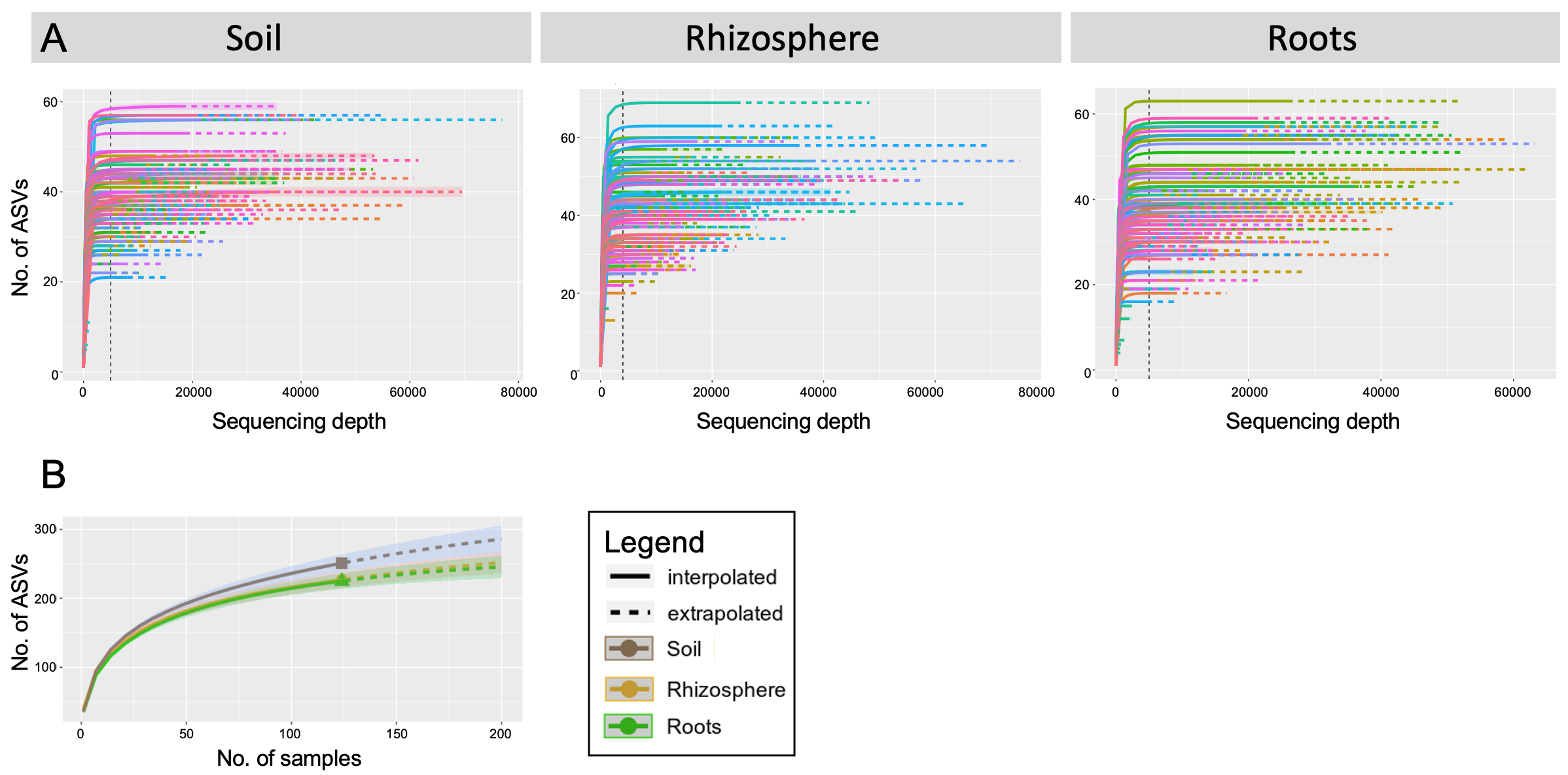


**Figure S3.** A) Sequencing-depth-base rarefaction and B) sample-base rarefaction (solid line segments) and extrapolation (dotted line segments) curves for soil, rhizosphere and root samples. Vertical dotted lines in A identify the sampling depth of 5000 sequences used for data normalization.

**
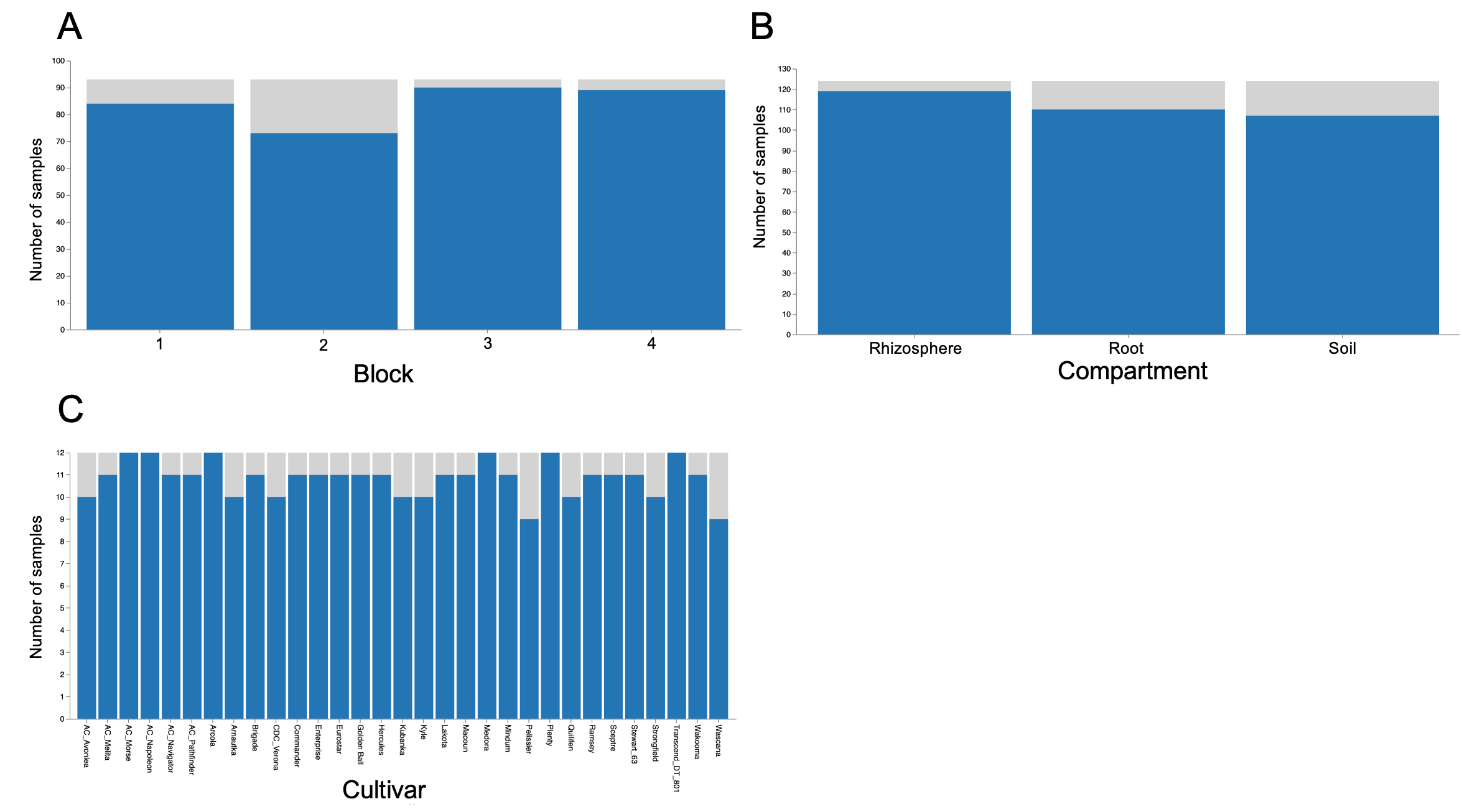
**

**Figure S4.** Number of retained samples per block (A), compartment (B), and cultivar (C) for a sampling depth of 5000 sequences per sample. A total of 1680000 (32.9%) sequences were retained in 336 (90.3%) samples at the specified sampling depth.

**Figure S5.** Provided as a separate file in pdf format. RAxML phylogeny showing the taxonomic assignment of each amplicon sequence variant (ASV, shown in blue) at the family (left) and genus levels (right). Phylogenetic trees on the right side represent the clades colored in grey in the phylogenetic tree on the left side. Only bootstrap values > 70 are shown. Within genus phylotypes have been determined using a clustering threshold of 99%.

**
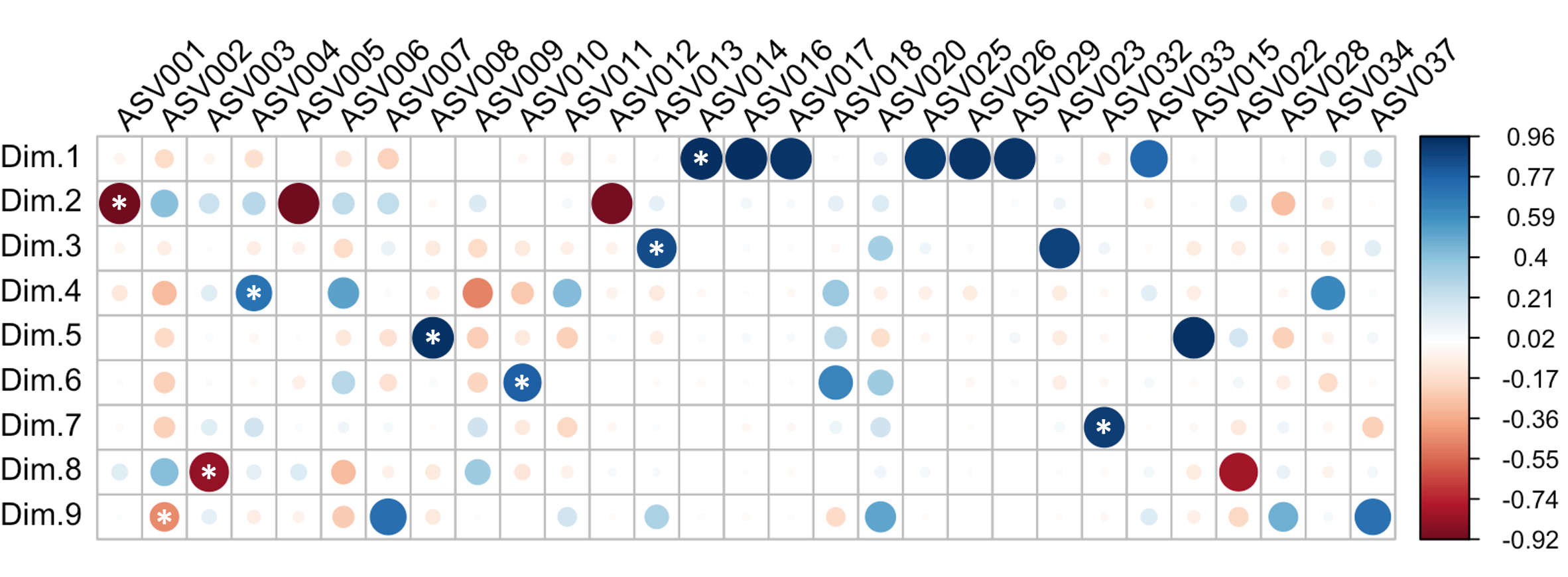
Figure S6.** Correlation (saturation coefficient, from -1 to +1) of the core ASVs (29) on the first nine principal components (dimension 1 to 9). Stars identify the ASVs selected on each dimension and for which the linear mixed model was run.


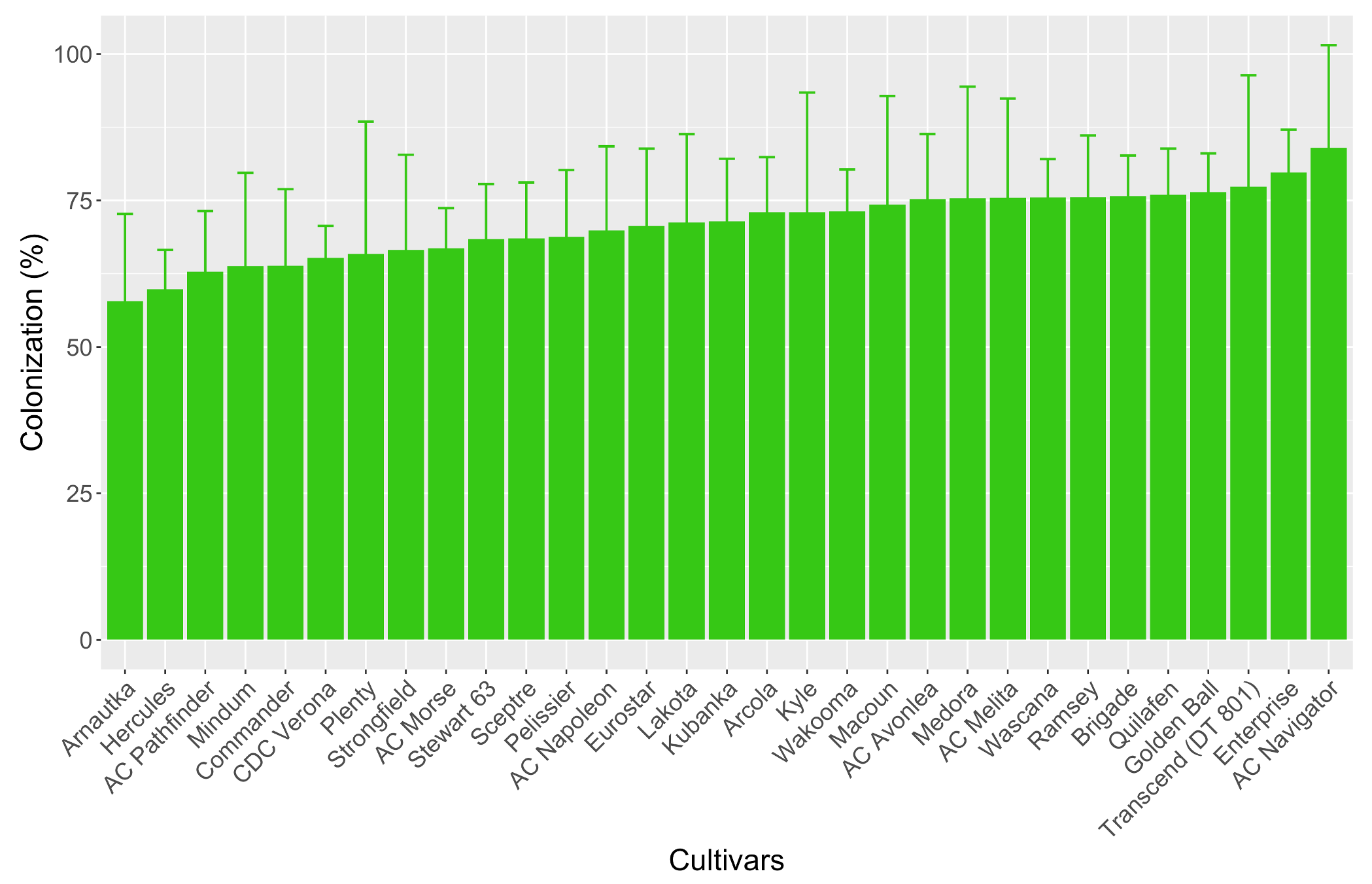
**Figure S7.** Root colonization measured for the five landraces (Arnautka, Kubanka, Mindum, Golden Ball, Pelissier) and 26 cultivars.
